# Identifying optimal conditions for precise knock-in of exogenous DNA into the zebrafish genome

**DOI:** 10.1242/dev.204571

**Published:** 2025-06-19

**Authors:** Sarah Oikemus, Kai Hu, Masahiro Shin, Feston Idrizi, Aliece Goodman-Khan, Amy Kolb, Krishna S. Ghanta, Jonathan Lee, Atish Wagh, Scot A. Wolfe, Lihua J. Zhu, Jonathan K. Watts, Nathan D. Lawson

**Affiliations:** ^1^ Department of Molecular, Cell, and Cancer Biology, University of Massachusetts Chan Medical School, Worcester, MA 01605, USA; ^2^ RNA Therapeutics Institute, University of Massachusetts Chan Medical School, Worcester, MA 01605, USA

**Keywords:** Zebrafish, CRISPR, Homology-dependent repair, Genome editing

## Abstract

CRISPR nucleases can be used to insert exogenous DNA into the zebrafish genome by homology-dependent repair (HDR), although germline transmission rates for precise edits remain quite low. Comparative studies to optimize HDR parameters for introducing base pair changes using short-read deep sequencing have been successful, but similar analysis for insertions is challenging due to read-length constraints. Here, we quantified editing outcomes using long-read sequencing to identify optimal template and CRISPR parameters for precise targeted insertion in zebrafish. Through side-by-side comparisons, we found that chemically modified templates out-perform those released *in vivo* from a plasmid, while Cas9 and Cas12a nucleases performed similarly for targeted insertion. Consistent with previous studies, precise editing rates were dependent on the distance between a double-strand break and the inserted sequence. We further found that non-homologous base pairs in homology templates significantly reduced precise editing rates. Using optimized parameters, we consistently achieved germline founder rates of greater than 20% for precise insertions across four loci. Together, our quantitative analyses identified optimal conditions for precise insertion of exogenous DNA into the zebrafish genome.

## Teaser

**Summary:** Quantitative side-by-side comparison of commonly used homology templates and CRISPR nucleases allows identification of optimal conditions for germline transmission of precise insertions in zebrafish.

## INTRODUCTION

The availability of programmable nucleases has enabled widespread application of reverse-genetic approaches in zebrafish (Richardson et al., 2023). The most common application is using a CRISPR nuclease (e.g. *Streptococcus pyogenes* Cas9) to induce double-strand breaks (DSBs) that lead to deleterious lesions predominantly due to the error-prone microhomology-mediated end-joining pathway (MMEJ; Gagnon et al., 2014; Hruscha et al., 2013; Thyme and Schier, 2016). Since a DSB can also stimulate homology-directed repair (HDR), introducing a homologous template can promote site-specific insertion of exogenous DNA (referred to as ‘knock-in’), albeit at very low rates in many species (Richardson et al., 2023). In zebrafish, CRISPR-induced HDR has been used to introduce disease-associated sequence variants, epitope tags, transcriptional and translational fluorescent protein reporters, and conditional knockout cassettes at numerous loci (Armstrong et al., 2016; Burg et al., 2018; de Vrieze et al., 2021; Eschstruth et al., 2020; He et al., 2020; Hisano et al., 2015; Luo et al., 2018; Prykhozhij et al., 2018; Shin et al., 2023; Tessadori et al., 2018; Wang et al., 2019; Wierson et al., 2020). Despite these advances, precise targeted knock-in remains highly variable and inefficient, while quantitative side-by-side comparisons across multiple loci are limited. Thus, further work to identify parameters for improvement is warranted.

A common variable across targeted knock-in studies is the type of HDR template. To introduce sequence variants, most studies rely on single-stranded oligodeoxynucleotides (ssODNs) for which conditions have been optimized to achieve acceptable founder rates (>5%) across many loci (Armstrong et al., 2016; Carrington et al., 2022; de Vrieze et al., 2021; Prykhozhij et al., 2018; Solman et al., 2022; Tessadori et al., 2018). To insert exogenous sequence, a range of different template types has been applied depending on the size of the transgene. For small inserts (e.g. epitope tags), most groups have used single- or double-stranded (ds) ODNs (Boel et al., 2018; Gagnon et al., 2014; Ghanta et al., 2021; Hruscha et al., 2013; Lavalou et al., 2019). In the case of HDR templates for larger reporter cassettes, most initial studies relied on circular plasmids or those linearized with a co-injected nuclease (Gillotay et al., 2020; He et al., 2020; Hisano et al., 2015; Hoshijima et al., 2016; Luo et al., 2018; Wierson et al., 2020). More recent studies have incorporated chemical modifications on synthetic templates or PCR primers prior to template amplification to improve targeted insertion in comparison to unmodified templates (DiNapoli et al., 2020; Ghanta et al., 2021; Krueger and Morris, 2022; Mi and Andersson, 2023; Perez-Rico et al., 2020; Zhang et al., 2023). These modifications are thought to improve HDR at the target site by reducing degradation or concatemerization of the template *in vivo* (Ghanta et al., 2021). In the case of reporter lines, screening injected embryos for fluorescent protein expression mitigates low rates of precise insertion to achieve high rates of germline transmission (Hisano et al., 2015; Mi and Andersson, 2023; Wierson et al., 2020). However, visual screening in these cases is limited to highly expressed genes. For inserts for which screening is not possible (e.g. short epitopes, loxP, attP sites), establishing lines can be inefficient and labor intensive, in part due to the prevalence of imprecise editing events that reduce the chance to obtain a functional germline allele (Hruscha et al., 2013; Ma et al., 2024; Prykhozhij et al., 2018).

Cas9 is the most common nuclease used in zebrafish studies, but other CRISPR nucleases that recognize distinct protospacer adjacent motif (PAM) sequences have also been used (Liang et al., 2022; Liu et al., 2019; Moreno-Mateos et al., 2017). *Lachnospiraceae* bacterium Cas12a (hereafter Cas12a) recognizes a 5′-TTTN PAM followed by a spacer of up to 24 nucleotides and generates a DSB that is 18 nt downstream on the same strand as its PAM, leaving a 5-nt 5′ overhang, unlike the blunt ends created by Cas9 (Zetsche et al., 2015). Given the long distance between a Cas12a-induced DSB and the PAM, initial deleterious repair events may not prevent subsequent spacer recognition and cutting at the target (Zetsche et al., 2015). The fact that small, imprecise edits in the Cas12a spacer may still be recut, coupled with the availability of a single-strand overhang, is thought to contribute to higher HDR rates stimulated by Cas12a at some loci in zebrafish and human cells compared to Cas9 (Moreno-Mateos et al., 2017; Shahbazi et al., 2019). However, use of alternative CRISPR nucleases remains limited in the zebrafish community, despite their commercial availability, and further quantitative analyses comparing Cas9 and Cas12a with respect to knock-in are lacking.

A challenge in identifying optimal conditions for targeted knock-in in zebrafish is how to measure repair events accurately in side-by-side comparisons across multiple loci. Assessing germline transmission is the ideal metric, but comparing multiple conditions and loci across replicate experiments is challenging due to tank space requirements, especially when precise repair events can be rare. For introducing single-nucleotide variants by knock-in, short-read deep sequencing on the Illumina platform to quantify somatic editing rates has been successfully applied to identify optimal parameters (de Vrieze et al., 2021; Prykhozhij et al., 2018). Importantly, these studies indicate that sequencing-based analysis of somatic editing rates in injected embryos can serve as a reasonable proxy for germline transmission frequency. However, similar analysis for targeted insertions has been limited (Ma et al., 2024), likely due to constraints on read length for Illumina sequencing libraries, which are 150 nucleotides for many platforms. Confirmation of precise knock-in events requires reliable sequencing reads encompassing the entire insert, including not only the insertion sequence but spanning beyond the regions of homology between the donor template and the genomic target site. For a standard Illumina library, a precise knock-in event would comprise most of the amplicon, while unmodified fragments, or those bearing small insertions or deletions (indels), would be much shorter. In these heterogeneous mixtures, larger fragment sizes amplify less efficiently than smaller fragments during PCR (Dabney and Meyer, 2012), while library purification steps and Illumina flow cell clustering further bias against longer DNA fragments (Bronner et al., 2014), leading to size bias in the resulting sequence. Finally, repair events from multiple insertions, or larger deletions that eliminate primer-binding sites would not be detected at all. Thus, Illumina short-read sequencing would not provide a reliable metric to quantify all potential HDR-mediated insertional end-products at the genomic target site. To address these issues, we applied single-molecule long-read sequencing on the Pacific Bioscience platform, which allows sequencing reads more than 10 kb in length (Eid et al., 2009), to quantify repair events accurately in zebrafish embryos. Using this approach, we compared several types of HDR templates using both Cas9 and Cas12a across five different loci to identify optimal parameters for targeted knock-in. We then used these optimized conditions to generate precise insertion alleles at four different loci, with founder rates of at least 20% in each case. Together, our observations define important parameters to consider for efficient introduction of precise targeted insertions into the zebrafish germline.

## RESULTS

### Choice of parameters to compare for precise knock-in

**Fig. 1.**
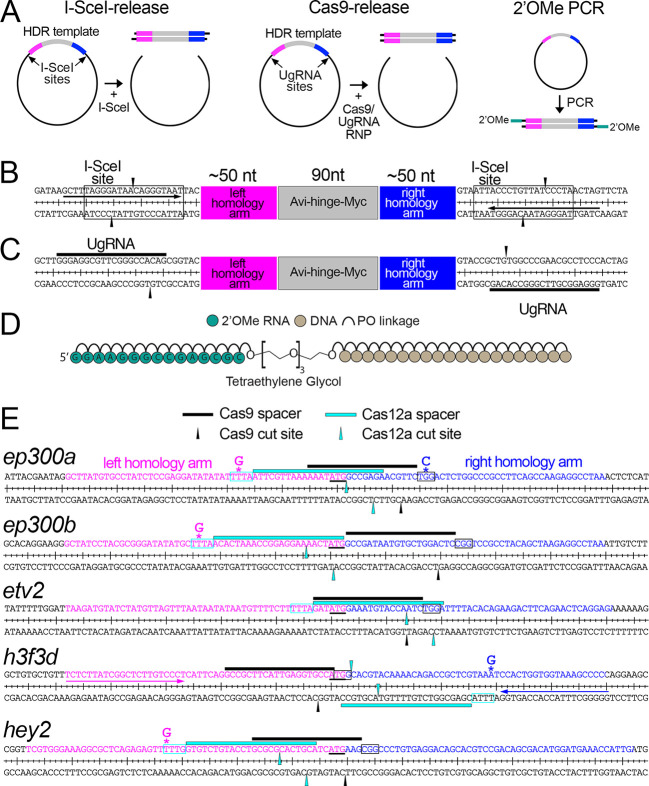
**Target genes and sequences.** (A) Schematic of linear HDR templates, released from plasmids by co-injection with I-SceI or Cas9, or amplified by PCR. (B,C) Plasmid vector backbones for *in vivo* release of HDR templates by I-SceI (B) or Cas9 (C) digestion. (B) I-SceI site is indicated by the box and cleavage sites indicated by black arrowheads. Arrows denote forward and reverse priming site for universal PCR primer. (C) UgRNA spacer sequence is indicated by black lines. The site of Cas9-induced DSB is shown by black arrowheads. (D) Schematic of the modified oligonucleotide primer used for PCR. PO, phosphodiester. (E) Target genes for HDR-mediated insertion. Black and cyan lines denote Cas9 and Cas12a spacer sequences, respectively. A line located above or below the sequence indicates CRISPR spacer target on the positive or negative strand, respectively. Boxes indicate the PAM. Black and cyan arrowheads denote the location of Cas9 and Cas12a cleavage sites, respectively. Note that Cas12a leaves a 5′ overhang. The start codon at each target is underlined. Asterisks indicate the location of PAM mutations engineered into HDR templates. Left and right homology arm sequences are magenta and blue, respectively. For *h3f3d*, forward and reverse gene-specific primers are indicated by magenta and blue arrows, respectively.

Our goal was to identify optimal conditions for precise targeted knock-in into the zebrafish genome through side-by-side comparisons of commonly used HDR templates and CRISPR nucleases. For HDR templates, we compared three types of linear double-stranded DNA templates (Fig. 1A). We tested plasmids for which a linear HDR template is released by plasmid digestion with a co-injected nuclease. This approach has been widely applied across numerous studies, with variation in types of nuclease and vector target sequence used for template release (Harish et al., 2023; Hoshijima et al., 2016; Julich and Holley, 2024; Keating et al., 2024; Liu et al., 2023; Shin et al., 2023; Spikol et al., 2024; Wierson et al., 2020). We chose to compare the use of I-SceI meganuclease and Cas9 in targeting vector-specific sequences flanking the HDR template (Fig. 1A). I-SceI is a yeast homing endonuclease that recognizes a non-palindromic 18 bp site that is not present in mammalian or teleost fish genomes (Fig. 1B; Jacquier and Dujon, 1985; Thermes et al., 2002). It has been used successfully to release linear DNA fragments from circular plasmids *in vivo* to increase both random genomic insertion and targeted knock-in rates in zebrafish (Hoshijima et al., 2016; Shin et al., 2023; Thermes et al., 2002). For Cas9-mediated release, we relied on a previously described spacer sequence referred to as a ‘universal guide RNA’ (UgRNA; Fig. 1A,C) that is not present in the zebrafish genome and was designed for optimal targeting by Cas9 (Wierson et al., 2019). For each case, we constructed vectors in which I-SceI sites or UgRNA target spacer and PAM sequences flank the HDR template (Fig. 1B,C). In parallel, we compared these plasmid-released templates to those generated by PCR using chemically modified primers, an approach used successfully in recent studies (Mi and Andersson, 2023; Zhang et al., 2023). For this purpose, we designed primers complementary to the vector I-SceI sites (Fig. 1B). These primers were used for all target templates in the initial analysis and yield similar non-homologous sequence at the ends of the HDR template as those released from plasmid backbones. For PCR, we synthesized primers with 2′-O-methyl-modified (2′OMe) ribose bases attached to the 5′ end of the deoxyoligonucleotide by a tetraethylene glycol (TEG) linker (Fig. 1D). 2′OMe end modifications should limit availability of an HDR template as a substrate for ligase IV or nucleases, reducing concatemerization and degradation, respectively. Accordingly, we have shown that 2′OMe-modified ODN templates form fewer concatemers and improve HDR rates compared to those that are unmodified (Ghanta et al., 2021). In addition to different template topologies, we compared Cas9 and Cas12a nucleases, the latter of which is thought to be better for stimulating HDR (Moreno-Mateos et al., 2017; Shahbazi et al., 2019).

To generate results that might be broadly applicable across most zebrafish loci, we introduced DSBs within 20 bp of the ATG for five genes (*ep300a*, *ep300b*, *etv2*, *h3f3d* and *hey2*) to insert an epitope tag in-frame with the start codon (Fig. 1E, Table S1). Our choice to target the ATG was arbitrary, but it allowed us to mimic cases for which the desired target site is highly constrained and requires precise edits on both ends; the gene products themselves do not otherwise have relevance to the outcomes of this study. To compare Cas9 and Cas12a, we identified spacer and PAM targets for each in close proximity (Fig. 1E). To generate HDR templates, we cloned gBlock fragments containing 50 bp homology arms flanking an Avitag-Myc epitope immediately downstream of the target start codon into the vectors described above (Fig. 1B,C). When possible, we designed HDR templates such that Avitag-Myc sequence would separate the PAM and spacer sequences in the event of precise insertion, thereby preventing re-targeting by the CRISPR nuclease. In cases for which this was not possible, we introduced point mutations (silent when in coding sequence) in the PAM in the template sequence (Fig. 1E).

### Identifying optimal parameters for precise knock-in using somatic editing rates

**Fig. 2.**
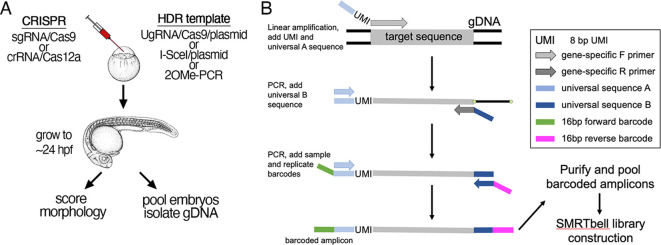
**Experimental design for quantifying somatic repair events.** (A) Schematic showing the steps to inject CRISPR RNPs and HDR templates at the one-cell stage, followed by scoring and isolation of genomic DNA. (B) Schematic describing the step-by-step construction of amplicon libraries for long-read sequencing. F, forward; R, reverse; UMI, unique molecular identifier.

We first determined optimal CRISPR ribonucleoprotein (RNP) complex doses for each target by assessing resistance to restriction enzyme digestion of sites near the DSB due to deleterious repair events as a proxy for nuclease activity in injected embryos (Fig. S1A). In each case, we chose the lowest RNP dose that caused complete or near-complete loss of restriction digestion (Fig. S1B, Table S1). Subsequently, we co-injected each type of CRISPR RNP at the optimal dose together with different HDR templates and, when appropriate, vector-targeted nucleases (I-SceI or Cas9/UgRNA RNP), into one-cell-stage zebrafish embryos (Fig. 2A). For HDR templates, we injected plasmid or PCR-amplified fragments at comparable molar amounts to ensure equal copy number. We also injected CRISPR RNP alone, or left embryos uninjected, as controls. Across all conditions, three different lab members performed injections to generate triplicate data points and mimic real-world application. At 24 hpf, we scored embryos as ‘normal’, ‘monster’ (non-specific morphological abnormalities) or ‘dead’ (completely degraded embryos without any obvious morphology). We subsequently pooled normal embryos for isolation of genomic DNA. In general, co-injection with any nuclease and HDR template together led to a modest decrease in survival compared to nuclease alone (Fig. S2, Table S2), although we did not observe significant differences between template types across all targets (Fig. S2).

To compare different templates and CRISPR nucleases, we applied deep sequencing to identify and quantify all somatic repair events at the target sites in zebrafish embryos at 24 hpf following injection at the one-cell stage. To ensure that we reliably captured all repair events, we performed single-molecule sequencing using the Pacific Biosciences (PacBio) platform, which allows for long read lengths (Eid et al., 2009). To minimize potential size bias during library preparation, we designed PCR primers to amplify fragments of at least 2 kb in length, in which case precise insertions would increase fragment length by less than 5%. For accurate quantification of repair events, we performed an initial linear amplification (LA) step with a single gene-specific primer containing a library of semi-random 8-bp unique molecular identifiers (UMIs) and a common universal adapter sequence (Fig. 2B). The incorporation of common universal primers allows for subsequent PCR amplification of multiple libraries together after mixing them into pools. This step also uniquely tags every primed DNA fragment in the initial mixture for accurate quantification. UMIs also allow us to filter out ‘jackpot’ events caused by some DNA species predominating due to PCR overamplification, which can skew quantification of repair events (Johnson et al., 2023; Salk et al., 2018). We subsequently PCR-amplified the UMI-tagged fragments using a reverse gene-specific primer that also contains a universal sequence distinct from that used in the first round of amplification. In successive rounds of PCR using universal primers, we then added barcode combinations to distinguish replicates and conditions (Fig. 2B). All libraries were then pooled and subjected to single-molecule sequencing.

**Fig. 3.**
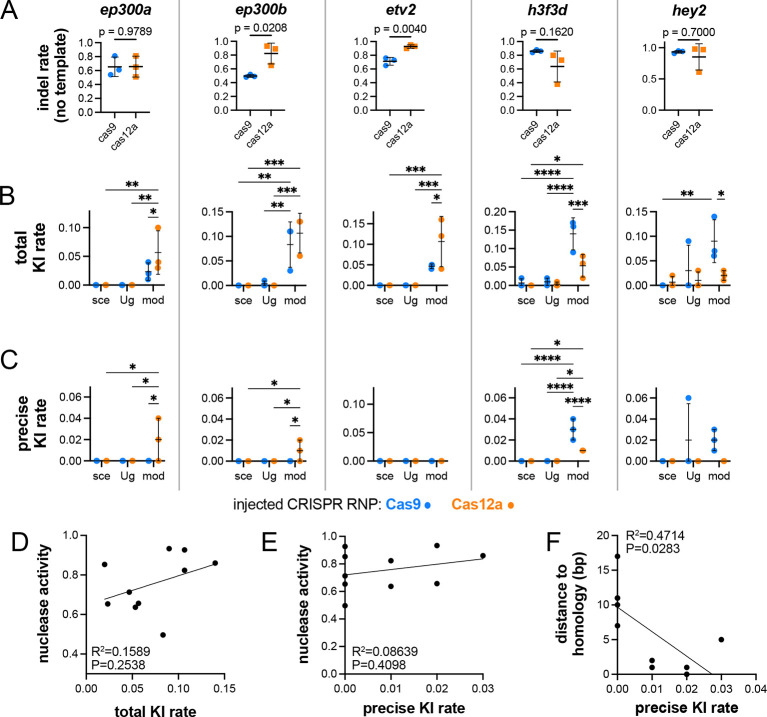
**Quantification and comparison of repair events with different HDR templates and CRISPR nucleases.** (A) Indel rates in embryos injected only with Cas9 or Cas12a targeting the indicated gene in the absence of HDR template. Unpaired *t*-test for all except *hey2* for which a Mann–Whitney test was used due to the non-normal distribution of this sample; *P*-values indicated. (B) Total KI rate at the indicated locus, considering any integration of the epitope tag sequence. (C) Precise KI rate. (B,C) Template type indicated on *x*-axis. mod, linearized template amplified by PCR with 2′OMe-modified primers; sce, plasmid linearized with I-SceI; Ug, plasmid linearized with Cas9 targeting universal vector sgRNA spacers. *P<0.05, **P<0.01, ***P<0.001, ****P<0.0001 (two-way analysis of variance, Šidák's multiple comparison test). In A-C, error bars represent s.d.; *n*=3. (D-F) Comparison of nuclease activity (indel rate in the absence of HDR template) and total KI rate (D), nuclease activity and precise KI rate (E) and distance from the cleavage site to start of exogenous sequence and precise KI rate (F).

We developed a computational pipeline to identify and quantify rates for the following repair events from our deep sequencing data: (1) ‘indel rate’ refers to the number of fragments without any evidence of exogenous transgene insertion, but otherwise bearing insertions or deletions at the target site, compared to all other fragments; (2) ‘total KI rate’ was calculated using the number of fragments containing any epitope insertion, including precise events as well as those with any other modification, including multiple inserts or indels within the inserted sequence or homology arms, compared to all other fragments; and (3) ‘precise KI rate’ was calculated from the number of unique fragments for which only precise insertions with seamless junction sites between HDR template and endogenous sequence were present versus all other counted fragments. We first quantified and compared nuclease activities by calculating indel rates for each nuclease without template. Across all loci indel rates ranged from 50% to 90% with both nucleases exhibiting comparable rates at common targets, except for *ep300b* and *etv2* for which Cas12a was significantly more active (Fig. 3A, Table S3). Most Cas9 indels were under 10 bp, while Cas12a induced larger local lesions (Fig. S3A,B, Table S4), consistent with previous observations (Kim et al., 2016; Moreno-Mateos et al., 2017). Deletions were usually the predominant lesion, except for *ep300b* and *etv2*, in which Cas9 also induced a large proportion of insertions (Fig. S3C), suggesting some degree of locus-dependent repair events. Cas12a generally induced a significantly lower proportion of insertions than Cas9 (Fig. S3A,C), similar to observations in human cells (Kim et al., 2016), and both nucleases also induced much larger deletions up to 1 kb, albeit at low frequencies (Fig. S3B, Table S4).

Despite robust nuclease activities across all targets, we observed very low and inconsistent total KI rates from templates released from plasmids with I-SceI or Cas9 targeting the UgRNA spacers (‘sce’ or ‘Ug’, respectively, in Fig. 3B; Table S3). For *ep300a* and *etv2*, we failed to detect any insertions from plasmid-released templates, while total KI rates well below 1% were generally found at the remaining targets, except for *hey2* for which average rates almost reached 10% (Fig. 3B, Table S3). By contrast, embryos injected with 2′OMe-modified templates consistently exhibited total KI events with rates averaging 2-14% across all loci (Fig. 3B). At three loci we noted significant but inconsistent differences between nucleases: Cas12a induced higher total KI rates at *etv2*, while Cas9 performed better at *h3f3d* and *hey2* (Fig. 3B). However, precise KI rates were much lower than total KI. Indeed, for plasmid-released templates, we observed precise KI events only in a single replicate at *hey2* (Fig. 3C). PCR-amplified 2′OMe-modified templates performed slightly better with precise KI detected at every target except *etv2*, although average rates were under 5% across all loci (Fig. 3C, Table S3). As with total KI, we noted differences between Cas9 and Cas12a for precise KI. For example, Cas9 failed to induce precise KI at *ep300a* or *ep300b* but performed better than Cas12a at *h3f3d* and *hey2* (Fig. 3C, Table S3). One possibility to explain this variation is simply difference in nuclease activity (Fig. 3A). However, we did not observe a significant correlation across all targets between nuclease activity and total or precise knock-in rates (Fig. 3D,E). Furthermore, average knock-in rates remained relatively similar across a broad RNP dose range at the *h3f3d* locus up to 10 fmol per embryo (Fig. S4, Table S5). While RNP activity may not explain observed differences in KI rates, we noted a significant and inverse correlation between precise KI rate and the distance from exogenous insert sequence to the CRISPR-induced DSB, with no precise editing events detected when this distance was more than five nucleotides (Fig. 3F). Taken together, our observations suggest that 2′OMe-modified linear templates provide a benefit over those released from plasmids for driving precise KI. We further find that precise insertion rates are highly sensitive to the distance between the exogenous insert and the location of the DSB. However, precise KI rates remained very low with several loci exhibiting no precise insertions.

### Homology-matched templates improve HDR-mediated insertion rates

During HDR-mediated DNA repair, strand invasion of a homologous donor is initiated by the 3′ end of a resected DSB, which anneals to its homologous sequence and primes subsequent DNA synthesis to repair the break (Liao et al., 2024; Wright et al., 2018). For the templates used above, the presence of non-homologous nucleotides could prevent priming and extension by DNA polymerases, or lead to the incorporation of the non-homologous sequences at the endogenous target. In either case, non-homologous nucleotides at the ends of the HDR template could contribute to lower precise KI rates. To determine whether this was the case, we synthesized gene-specific 2′OMe-modified primers for PCR amplification of the *h3f3d* KI template (Fig. 1E) and used these to amplify a homology-matched HDR template. We then performed side-by-side injections to compare a homology-matched template with that amplified using the same vector-specific primers used above (see Fig. 1B). In this case, we performed injections only using Cas9 as the RNP since that performed better for precise KI than Cas12a at the *h3f3d* locus (Fig. 3C). As above, we applied LA-PCR and PacBio sequencing to quantify somatic repair events. In addition, we grew sibling embryos to adulthood to determine the rates of germline transmission for all editing events.

**Fig. 4.**
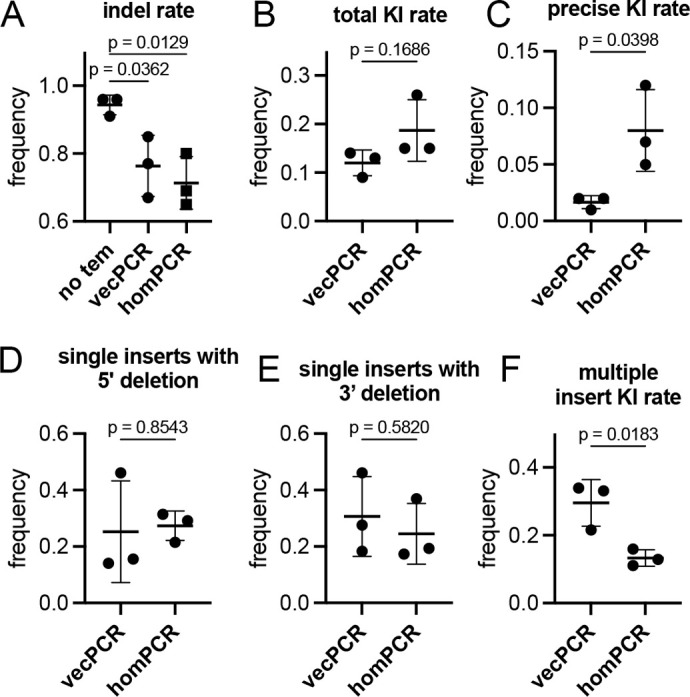
**Homology-matched HDR template improves precise knock-in rates.** (A) Indel rates with Cas9 RNP alone (no tem), or co-injected with HDR templates targeting the *h3f3d* ATG that were PCR-amplified with vector (vecPCR) or homology-matched (homPCR) primers. (B-F) Frequencies of targeted insertion of indicated repair event using vector or homology-matched template at the *h3f3d* locus. *P*-values are indicated (unpaired *t*-test). Error bars represent s.d.; *n*=3.

In injected embryos, indel rates were comparable in the presence of either template, but generally reduced compared to no template, likely due to a shift from MMEJ to HDR-mediated repair (Fig. 4A, Table S5). Overall knock-in rates appeared slightly increased with the homology-matched template, but this difference was not significant (Fig. 4B, Table S5). We observed a significant fivefold increase in precise knock-in rate when comparing homology-matched template to that amplified using vector primers (Fig. 4C, Table S5). At the same time, we observed a concomitant decrease in multiple tag insertions, while imprecise edits from indels at the 5′ or 3′ ends of integrated single tags were unchanged (Fig. 4D-F, Table S5). Consistent with rates of somatic repair events, we observed robust transmission of precise insertions at *h3f3d* in the germline of more than 20% of injected parental founder (P0) fish using a homology-matched template amplified by PCR with 2′OMe modified primers (Table 1). By contrast, we failed to obtain a single founder with a precise insertion at *h3f3d* when using a similarly amplified template with non-homologous nucleotides at the ends, despite germline transmission of indels and modified knock-in alleles (i.e. tag insertions that otherwise bear indels; Table 1). These observations suggest that non-homologous nucleotides on the ends of templates may interfere with HDR, significantly reducing germline transmission of precise knock-in alleles.

**Table 1.** Founder transmission and mosaicism of precise inserts

Gene	Template	Total numbers						
		Total P0	Indel*	5′ KI^‡^	3′ KI^‡^	Precise	Precise (%)	Founder mosaic (%)
*h3f3d*	hmPCR	30	27	20	16	7	23%	N.D.
*h3f3d*	vecPCR	30	23	9	7	0	0%	–
*foxc1a*	homPCR	15	N.D.	N.D.	N.D.	3	20%	20/50/82%
*prox1a*	homPCR	8	N.D.	N.D.	N.D.	2	25%	N.D.
*rasa1a*	Alt-R dsODN	10	N.D.	N.D.	N.D.	2	20%	45/42%

All injections were performed using a Cas9 RNP.

*Indels determined by loss of restriction digestion at the target in F1 progeny embryos. ^‡^Assessed by PCR with anchored Avitag primer and 5′ or 3′ gene-specific primer.

homPCR, homology-matched PCR primer amplified; N.D., not determined; vecPCR, vector PCR primer amplified.

**Fig. 5.**
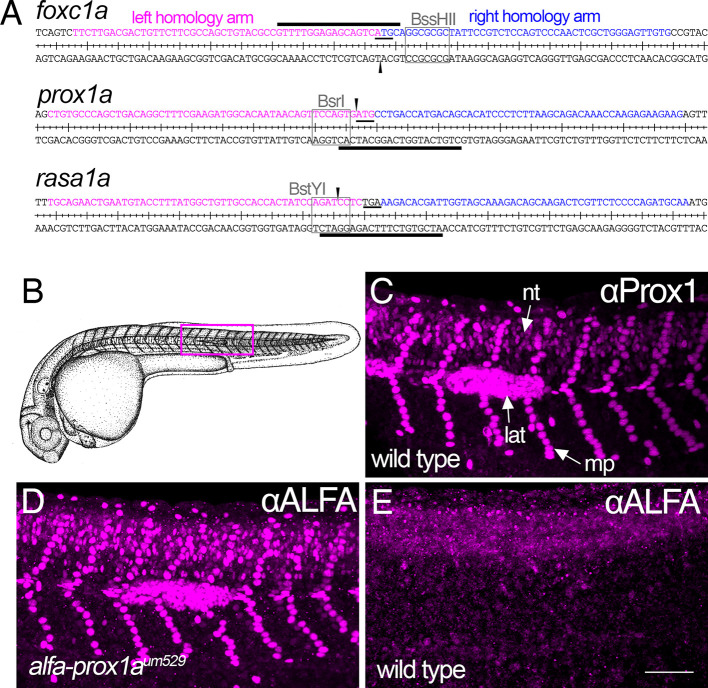
**Targeted insertion of an ALFA tag using optimized knock-in parameters.** (A) Sequence and flanking target insertion sites for *foxc1a*, *prox1a* and *rasa1a*. Left and right homology arms are indicated by magenta and blue, respectively. Black lines denote the Cas9 spacer sequence (line above or below sequence denotes positive or negative strand, respectively) and arrowheads mark the DSB sites. Start or stop codons are underlined. Restriction enzyme sites used to estimate nuclease activity are indicated by boxes and labeled. (B) Camera lucida of a zebrafish embryo at 31 hpf. Reproduced with permission from Kimmel et al. (1995). (C-E) Whole-mount immunostained embryos imaged by confocal microscopy; lateral views, anterior to the left, dorsal is up. (C) Wild-type embryos immunostained with polyclonal Prox1 antibody. Arrows denote fluorescence in neural tube (nt), lateral line primordium (lat) and muscle pioneers (mp). (D,E) Immunostaining of *alfa-prox1a^um529^* heterozygous (D) and wild-type sibling (E) embryos with a nanobody against ALFA. Scale bar: 80 µm.

To determine whether the optimized conditions identified here are generally applicable to other loci, we chose three additional genes to target: *foxc1a*, *prox1a* and *rasa1a*. For an insert in these cases, we used the ALFA epitope tag, a short amino acid sequence optimized for applications in model organisms and for which a high-affinity nanobody has been developed (Gotzke et al., 2019). We sought to insert the ALFA tag in-frame of the start codons of *foxc1a* and *prox1a*, or immediately upstream of the stop codon for *rasa1a* using Cas9 spacer sequences to induce a DSB within 5 bp from the tag insertion point (Fig. 5A). In all cases, HDR-mediated insertion of the ALFA sequence would separate the Cas9 PAM and spacer sequences, obviating the need to alter any sequences in the homology arm to prevent recutting. For *foxc1a* and *prox1a*, we constructed plasmids containing homology arm sequence flanking the ALFA tag in-frame with the start codon and synthesized 2′-OMe-modified primers to amplify a homology-matched template. For *rasa1a*, we used an Alt-R dsODN that bears proprietary chemical end modifications and is available commercially from Integrated DNA Technologies (IDT) (Fig. 5A, Table S6).

As above, we identified the optimal RNP dose by assaying loss of restriction enzyme digestion near the DSB due to deleterious repair events in injected embryos (for an example, see Fig. S5A). Using optimized RNP doses, we first confirmed knock-in at the target site in individual F0 embryos following co-injection with PCR-amplified or Alt-R HDR templates. For *foxc1a*, we assessed knock-in by digesting PCR amplicons using a restriction enzyme site in the ALFA tag sequence with 70% of embryos showing evidence of KI (Fig. S5A). For *prox1a* or *rasa1a*, we performed PCR using primers anchored in the ALFA tag sequence, with 30% and nearly all embryos exhibiting KI, respectively (Fig. S6B,C). Subsequently, we repeated injections and grew embryos to adulthood, followed by founder identification through PCR analysis of F1 progeny from individual outcrosses. For each target gene, we identified precise germline insertions in at least 20% of P0 fish (Table 1). Moreover, we generally observed low germline mosaicism from identified founders, with 20-80% of F1 embryos bearing a precise insertion (Table 1). Finally, to demonstrate the utility of the inserted epitope tag, we performed immunostaining using a nanobody recognizing the ALFA sequence on *alfa-prox1a^um529^* embryos, which bear a precise insertion at the ATG in the *prox1a* locus. At 24 hours post-fertilization (hpf), immunostaining with a polyclonal antibody against Prox1 normally detects nuclear-localized protein in muscle pioneer cells in the somites, as well as cells within the migrating lateral line primordium and neural tube (Fig. 5B,C). Whole-mount immunostaining with the ALFA nanobody on heterozygous F1 *alfa-prox1a^um529^* embryos revealed a pattern of expression identical to that detected with Prox1 polyclonal antibody (Fig. 5D), while wild-type siblings exhibited only background signal (Fig. 5E). Taken together, these results demonstrate that our optimized knock-in parameters can consistently yield high rates of precise insertion events in the zebrafish germline.

## DISCUSSION

Our quantitative sequence-based analysis of side-by-side comparisons across multiple target loci has allowed us to define optimized parameters for targeted knock-in in zebrafish. Together, these optimized conditions allowed us to consistently achieve founder rates of 20% or higher, making it straightforward and efficient to generate knock-in zebrafish lines where pre-screening is not possible. Several points of emphasis for improving precise knock-in rates emerged from our direct side-by-side comparisons. For the benefit of other groups in the zebrafish community, we have included an outline of steps and considerations we currently use to generate knock-in lines (see supplementary Materials and Methods).

Among the important parameters for efficient precise targeted knock-in is the type of HDR template. Most published studies have relied on linear fragments released from plasmids or generated by PCR, with a recent trend of using chemically modified primers. However, direct comparisons between template types have been lacking. In our side-by-side analysis, we consistently found that linear fragments containing 2′OMe end-modifications clearly outperformed plasmid-released templates across multiple targets. This finding is largely consistent with previous studies demonstrating that chemical modifications of ODN templates can lead to improvements in precise editing for short inserts or nucleotide changes (Ghanta et al., 2021; Krueger and Morris, 2022; Perez-Rico et al., 2020; Prykhozhij et al., 2018). Although our results are limited to short inserted sequences, recent studies have shown similar results with much longer reporter cassettes (Mi and Andersson, 2023; Zhang et al., 2023). As noted above, chemical end-modifications likely reduce template concatemerization and degradation. Use of a PCR-amplified or synthetic template also provides an easy way to fully match homology arm sequence to the target. Since homologous recombination typically requires 3′ annealing to prime repair strand extension, non-homologous sequences could hinder this process leading to alternative repair mechanisms that incorporate unmatched sequence at the target. Thus, non-homologous base pairs likely contribute to the low and inconsistent knock-in rates we observed for all templates in our initial comparison tests.

We observed that the distance between the DSB and the point of insertion for exogenous sequence was an important factor across all targets, consistent with previous studies in zebrafish (Prykhozhij et al., 2018; Tessadori et al., 2018) and in human induced pluripotent stem cells (Paquet et al., 2016). However, zebrafish appear more sensitive to this variable than human cells with no precise editing events detected for any distances longer than 5 nt between cut sites and homology start, even for highly active nucleases otherwise capable of stimulating knock-in events. This observation may simply reflect the generally higher rate of HDR mediated repair in human induced pluripotent stem cells than in zebrafish (Paquet et al., 2016). Notably, differences between precise insertion rates stimulated by Cas9 versus Cas12a in our study were likely due to the location of the DSB relative to the insert, rather than a functional distinction between the nucleases themselves. We did not otherwise observe a general trend for Cas12a to more efficiently induce HDR, as would be expected by previous work (Moreno-Mateos et al., 2017; Shahbazi et al., 2019). We recommend taking this into account when planning the design for a knock-in allele, using whichever nuclease might provide a DSB closer to the insertion point. Furthermore, the use of Cas9 variants with alternative PAM requirements (Friedland et al., 2015; Kleinstiver et al., 2015) should allow additional options to minimize the distance between the DSB and insert at nearly all sites. Since several different CRISPR nuclease variants are now commercially available (see supplementary Materials and Methods), nuclease choice should be a primary consideration in planning zebrafish knock-in experiments.

The zebrafish has continued to provide an ideal model for investigating developmental processes. As genome-editing approaches have matured, the zebrafish has also been increasingly used to model monogenic human disease, for example through the introduction of homologous disease-associated point mutations in orthologous zebrafish genes. Given the growing spectrum of candidate alleles for human genetic disease, highly efficient introduction of point mutations and exogenous sequence into the zebrafish genome is essential. Our findings here, based on quantitative side-by-side comparisons, outline a set of conditions and considerations for achieving efficient germline knock-in in zebrafish. In addition, our results underscore the benefit of applying stringent long-read deep sequencing to assess repair events accurately. We hope that future optimization studies will rely on similarly quantitative means to improve conditions further for targeted manipulation of the zebrafish genome.

## MATERIALS AND METHODS

### Zebrafish line maintenance

Zebrafish were maintained and bred according to protocols approved by University of Massachusetts Chan Medical School Institutional Animal Care and Use Committee. EK and EK2 wild-type lines were used for microinjections, outcrossing for founder identification, and establishing lines. All lines generated in this study are available upon request.

### CRISPR and donor design

We identified target sequence for *S. pyogenes* Cas9 and *Lachnospiraceae* bacterium Cas12a nuclease at the ATG of the *ep300a*, *ep300b*, *etv2*, *h3f3d* and *hey2* genomic loci (Table S1) using CHOPCHOP (Labun et al., 2019). A single Cas9 and Cas12a target site was chosen for each loci, with preference given to higher GC content over distance to ATG for Cas9 targets. Alt-R Cas9 cRNAs were synthesized by IDT and duplexed to Alt-R tracrRNA (IDT) according to the manufacturer's instructions. We prepared Cas12a crRNA templates by PCR using a target-specific oligonucleotide and T7 scaffold primer (5′-CTAATACGACTCACTATAGGGTTTCAAAGATTAAATAATTTCTACTAAGTGTAGAT-3′) and crRNAs were synthesized using the T7 MEGAscript kit (Thermo Fisher Scientific, AM1333), as previously described (Liu et al., 2019). Editing activity was confirmed by injection in the absence of repair template followed by PCR and enzyme digestion (Tables S1, S6). To generate HDR templates, two separate backbone vectors were generated: one with I-SceI sites and one with UgRNA (Wierson et al., 2020) flanking a SnaBI restriction site in both cases (Fig. 1). For this purpose, complementary single-strand oligonucleotide containing the I-SceI and UgRNA target spacer and PAM sequence were synthesized by IDT, annealed, and inserted via ligation-medated cloning into pBSSK− digested with SpeI and HindIII. For each target, we designed gBlocks (IDT) containing 50 bp homology arms and the Avi-myc tag sequence. The gBlocks were cloned into the SnaBI site of the I-SceI and UgRNA vector backbones using standard ligation. All plasmid donor sequences were confirmed by Sanger sequencing. Release of the repair template sequence was confirmed by *in vitro* digest with I-SceI or Cas9. Donor plasmids were phenol-chloroform extracted and ethanol-precipitated prior to injection. We generated linear double-stranded HDR templates by PCR amplification using 25 pg input plasmid, 4 µM final 2′OMe primers and Phusion High-Fidelity Polymerase (New England Biolabs; 98°C 10 s; 60°C 15 s; 72°C 10 s for 34 cycles). PCR fragments were gel purified using the ZymoDNA Clean and Concentrator Kit (Zymo Research) following the manufacturer's instructions. For *foxc1a*, *prox1a* and *rasa1a*, we identified Cas9 spacer targets manually that would give the closest DSB to the insertion site and used Alt-R Cas9 and sgRNAs as above. For *foxc1a* and *prox1a*, we cloned HDR template sequences by direct cloning of blunt-ended gBlocks into pJET1.2 (Thermo Fisher Scientific). Following sequence validation, we PCR-amplified linear HDR templates from plasmid using 2′OMe-modified forward and reverse gene-specific primers (Fig. 5A, Table S6). For *rasa1a*, we ordered ssODNs (Fig. 5A, Table S6) comprising the HDR template cassette containing Alt-R modifications (IDT) and annealed these prior to injection. All plasmids constructed in this study are available upon request.

### Oligonucleotide synthesis

For PCR amplification to generate linear HDR templates, RNA-TEG-modified oligonucleotides were synthesized using standard phosphoramidite-based methods on a Dr. Oligo 48 synthesizer with reagents from ChemGenes. The coupling times for 2′OMe-RNA and TEG spacer phosphoramidites were extended to 5 min. Oligonucleotides were deprotected by incubating in concentrated aqueous ammonia at 55°C for 16 h, followed by desalting using 3 kDa-cutoff Amicon ultrafiltration cartridges. Oligonucleotides were characterized with electrospray ionization on an Agilent 6530 Q-TOF LC/MS system.

### Microinjections

Zebrafish embryos obtained from EK wild-type in-crosses were used for one-cell-stage microinjections of RNPs and donor DNAs. Cas9/RNP complexes were prepared by incubating Alt-R SpCas9 nuclease V3 (IDT) with cRNA and tracrRNA at 37°C for 5 min before adding donor plasmid and the appropriate enzyme for release. For co-injections with templates released from plasmids, the injection mixture contained final concentrations of 5 ng µl^−1^ of plasmid DNA and either 1 unit µl^−1^ I-SceI enzyme or 5 µM Cas9/UgRNA RNP. Injection mixtures also contained target CRISPR RNP at final concentrations to give the ‘per embryo’ amounts shown in Table S1. For plasmid-released templates, we injected a volume of 2 nl, which results in 10 pg of template DNA per embryo. For PCR-amplified templates, injection mixtures contained target CRISPR RNP (see Table 1 for optimized RNP amounts) and linear template at a final concentration of 1.25 ng/µl, of which 2 nl was injected to give 2.5 pg per embryo. Recombinant Cas12a was purified as described previously (Liu et al., 2019). Cas12a RNP complexes were prepared by incubating recombinant LbCas12a and crRNA at room temperature for 20 min before addition of donor plasmid and enzyme. All injections were made into the early one-cell blastomere and every effort was made to avoid injection into the yolk. We first identified optimal RNP doses using injections with CRISPR RNPs in the absence of template for each target (Fig. S1, Table S1) and used these for subsequent studies, except for the *h3f3d* dose-response experiment. We incubated Cas9-injected embryos overnight at 28.5°C. Embryos injected with LbCas12a were incubated at 32°C for 4 h immediately following injection (Liu et al., 2019; Moreno-Mateos et al., 2017), then placed at 28.5°C overnight. Injections were performed in three independent replicates. We assessed toxicity at approximately 24 hpf by scoring numbers of normal, malformed (monster) and dead embryos.

### Library construction and PacBio sequencing

Genomic DNA was extracted from 25 injected embryos at approximately 24 hpf using the QIAGEN DNeasy Blood and Tissue kit. We generated target-specific amplicons of at least 2 kb by linear amplification and PCR. Briefly, we first performed linear amplification using a single forward gene-specific primer at least 1 kb upstream of the ATG containing UMIs (8-bp semi-random barcodes to avoid redundant counting; Table S6) and a 5′ universal anchor sequence (Fig. 2B, Table S6) with 25 ng of genomic DNA and Q5 high fidelity polymerase (NEB) under the following conditions: 98°C, 15 s; 62°C, 25 s; 72°C 45 s for ten cycles. To this reaction, we added a forward universal A primer and reverse gene-specific primer anchored at least 1 kb downstream of the ATG containing universal B sequence (Fig. 2B, Table S6) at a final concentration of 500 nM followed by 20 cycles with the conditions listed above. We next used 0.5 µl of the resulting reaction in a fresh PCR amplification using universal A forward and universal B reverse primers each containing barcodes for experimental conditions or replicate (Fig. 2B, Table S6) using Q5 polymerase (NEB; 98°C, 15 s; 62°C, 25 s; 72°C 45 s for ten cycles). Products were pooled in equal amounts based on agarose gel quantification using Fiji and purified using AMPure PB Beads (Beckman Coulter) following the manufacturer's instructions. Final library concentration was determined using a Qubit Fluorometer (Invitrogen), diluted to 20 ng/µl and submitted to the PacBio Core Enterprise at University of Massachusetts Chan Medical School for construction of a SMRTBell fragment library. For each library set, all libraries were pooled onto a single SMRT Cell and subjected to 30 h data collection to generate high fidelity circular consensus sequence (CSS) files.

### Sequence analysis

Barcoded sequences from CSS files were demultiplexed with Pheniqs (Galanti et al., 2021) and analyzed using two Nextflow-based analytical pipelines, which integrated in-house scripts and third-party tools. The first pipeline, sikipipe (https://github.com/hukai916/sikipipe), was designed for preprocessing, while the second pipeline, sikiclass (https://github.com/hukai916/sikiclass), was developed for downstream variant classification. Detailed description for both pipelines, including major steps, input and output specifications, and usage instructions, is available in their respective GitHub repositories. For preprocessing (using sikipipe), raw PacBio sequencing reads from different lanes (if applicable) were first merged into a single file. Reads in opposite orientations were then reverse complemented. Abnormal reads were identified and filtered out using a custom Python script (prep_data.py), with the following criteria: (1) read lengths outside the range 1000-5000 bp, or (2) reads for which the first 30 bases deviated significantly from the expected universal primer sequence. Universal primers were subsequently trimmed using Cutadapt (Martin, 2011) and read quality was accessed using FastQC (https://www.bioinformatics.babraham.ac.uk/projects/fastqc/). Next, UMIs were extracted and appended to the read names using UMI-tools (Smith et al., 2017). Reads were then grouped by UMI and collapsed at various cutoffs. The most frequent read in each UMI group was selected as the representative read for that group using a custom Python script (umi_correct.py). Throughout the preprocessing, statistics regarding the number of reads at each step and the read length distribution within each UMI group were generated for quality control. For variant classifications (using sikiclass), the first step involved categorizing the UMI-collapsed reads based on the presence of insert tag fragments. Each individual read was treated as the reference, and the insert tag was aligned to it using BWA (Li and Durbin, 2009). The resulting BAM files were then parsed with a custom Python script (parse_bam_tag.py) to categorize the reads into three classes: ‘without tag’, ‘with single tag’ and ‘with multiple tags’. Next, reads classified as ‘with single tag’ were further analyzed to identify potential indels. In this step, reads were first mapped to the expected references using Minimap2 (Li, 2018). The resulting BAM files were then processed with a custom Python script (parse_bam_indel.py) to locate indels, and the reads were further classified into subgroups including: ‘with precise tag’, ‘with 5′ indel’ and ‘with 3′ indel’, based on the coordinates of the indels. The sizes and positions of the indels along the reference sequence were also reported. For the ‘with precise tag’ reads, further classification was performed based on a single nucleotide polymorphism site using the script parse_snp.py. Reads classified as ‘with multiple tags’ were treated similarly to those ‘with single tag’ in terms of indel detection, but they were additionally filtered to exclude ‘natural indels’, those also identified in control samples, using the script filter_control_indel.py. Finally, read counts and sequences were reported after each processing step. For a more detailed description of the classification workflow, please refer to the method diagram available in the GitHub repository.

### Germline transmission

To identify *h3f3d* founders 30 injected P0 fish were screened for each condition by outcrossing to wild-type fish. For *foxc1a*, *prox1a* and *rasa1a*, the number of screened P0 fish is shown in Table 1. Genomic DNA was prepared from 30 embryos at 24 hpf using the QIAGEN DNeasy Blood and Tissue kit. Integration was assessed by PCR using primers outside of the homology region. Fragments corresponding to the correct size were cloned and sequenced to determine precise versus imprecise integration. Confirmed founders were outcrossed a second time and the percentage of germline transmission of the knock-in allele to F1 progeny was determined by screening at least 25 individual embryos by PCR.

### Whole-mount immunostaining

To detect endogenous or ALFA-tagged Prox1a, embryos were fixed with 8% paraformaldehyde in PBS overnight at 4°C, quenched with 3% H_2_O_2_ in methanol and stored in methanol at −20°C until use. Embryos were permeabilized sequentially with water for 30 min and 1× PBS containing 0.1% Tween 20 (PBSTw) and 1% Triton X-100 for 30 min at room temperature. For antigen retrieval, embryos were incubated with 0.1 M sodium citrate dehydrate containing 0.05% Tween 20, pH 6.0 for 20 min at 98°C. The following antibodies and dilutions were used: sdAb/ALFA-HRP (1:10,000; N1505-HRP, RRID:AB_3075989, NanoTag Biotechnologies), anti-Prox1 polyclonal rabbit antibody (1:1000; 11-002, RRID:AB_10013720, AngioBio) in blocking buffer (PBSTw containing 0.1% Triton X-100/2% DMSO/5% bovine serum albumin/1% goat serum). For detection of endogenous Prox1, embryos were incubated with Prox1 antibody in blocking buffer overnight at 4°C followed washing in PBSTw for 20 min at room temperature six times, then incubated with goat anti-rabbit IgG(H+L)-HRP (1:1000; ab6721, RRID:AB_955447, Abcam) overnight at 4°C. For detection of ALFA-Prox1a, embryos were incubated with sdAb/ALFA-HRP in blocking buffer overnight at 4°C and washed as above with PBSTw. For both antibodies, embryos were briefly washed with PBSTw, then 10% DMSO in PBS, and incubated with 1:50 TSA Plus tyramide-Cy3 in 1× Plus amplification diluent (NEL744001KT, Akoya Biosciences)/10% DMSO for detection. Embryos were imaged using an LSM 900 confocal microscope (Zeiss) and vertical projections were generated using Fiji (RRID:SCR_002285).

## Supplementary Material



10.1242/develop.204571_sup1 Supplementary information

Table S1. Description of targets, CRISPR doses and associated information.

Table S2. Raw viability and toxicity data.

Table S3. Long-read sequencing-based quantification of repair events comparing CRISPR nuclease and HDR templates.

Table S4. Distribution of indels in the absence of HDR template.

Table S5. Long-read sequencing-based quantification of repair events at the *h3f3d* locus.

Table S6. Oligonucleotide sequences used in this study.
