# Supplementary material for: Identifying optimal conditions for precise knock-in of exogenous DNA into the zebrafish genome
Source: Development. 2025 Jun 19;152(12):dev204571. doi: 10.1242/dev.204571 (PMC12211562; doi:10.1242/dev.204571)
Supplement: Supplementary information [file develop-152-204571-s1.pdf]

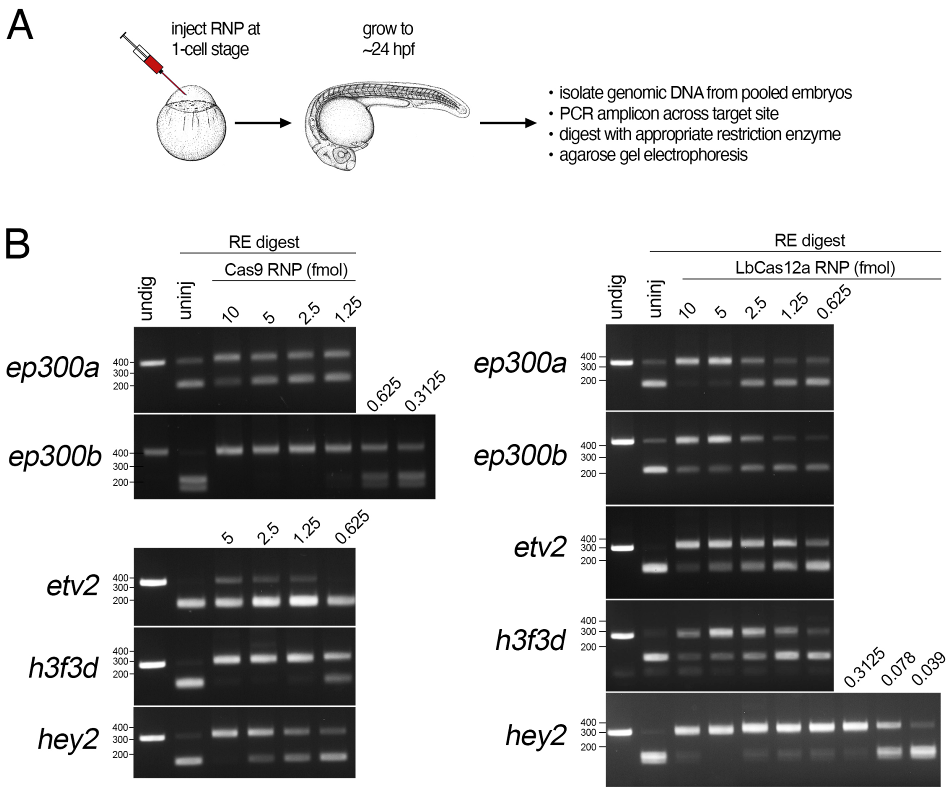

Fig. S1. Identifying optimal doses for Cas9 and Cas12 ribonucleotide (RNP) complexes. **A.** Schematic of injection strategy for optimizing RNP doses. **B.** Images of agarose gels showing amplified PCR products from indicated target. CRISPR RNPs and doses are indicated for each target. See Table S1 for restriction enzyme information and optimal doses used in the subsequent studies.

Development • Supplementary information

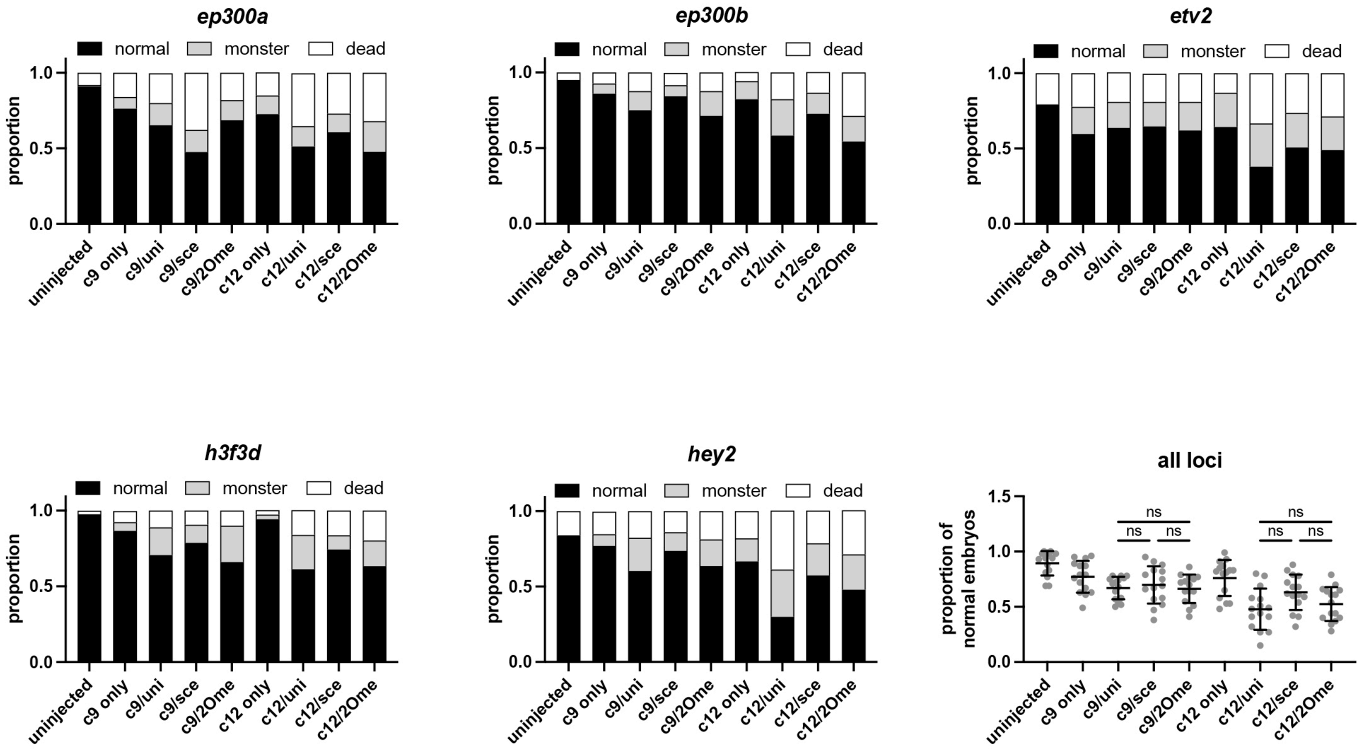

Fig. S2. Viability of injected embryos. Graphs showing proportion of embryos with indicated injection combination or left uninjected. Target locus is indicated for each panel. Data were compiled from three replicates and used to calculate proportions. Morphology was scored at 24 hours post fertilization. See Table S2 for raw data. "Monster" refers to any embryo with abnormal morphological appearance. c9 – cas9, c12 – cas12a, uni – template released from plasmid with Cas9/UgRNA RNP, sce – template released from plasmid with I-SceI, 2Ome – template PCR amplified with 2’OMe modified primers.

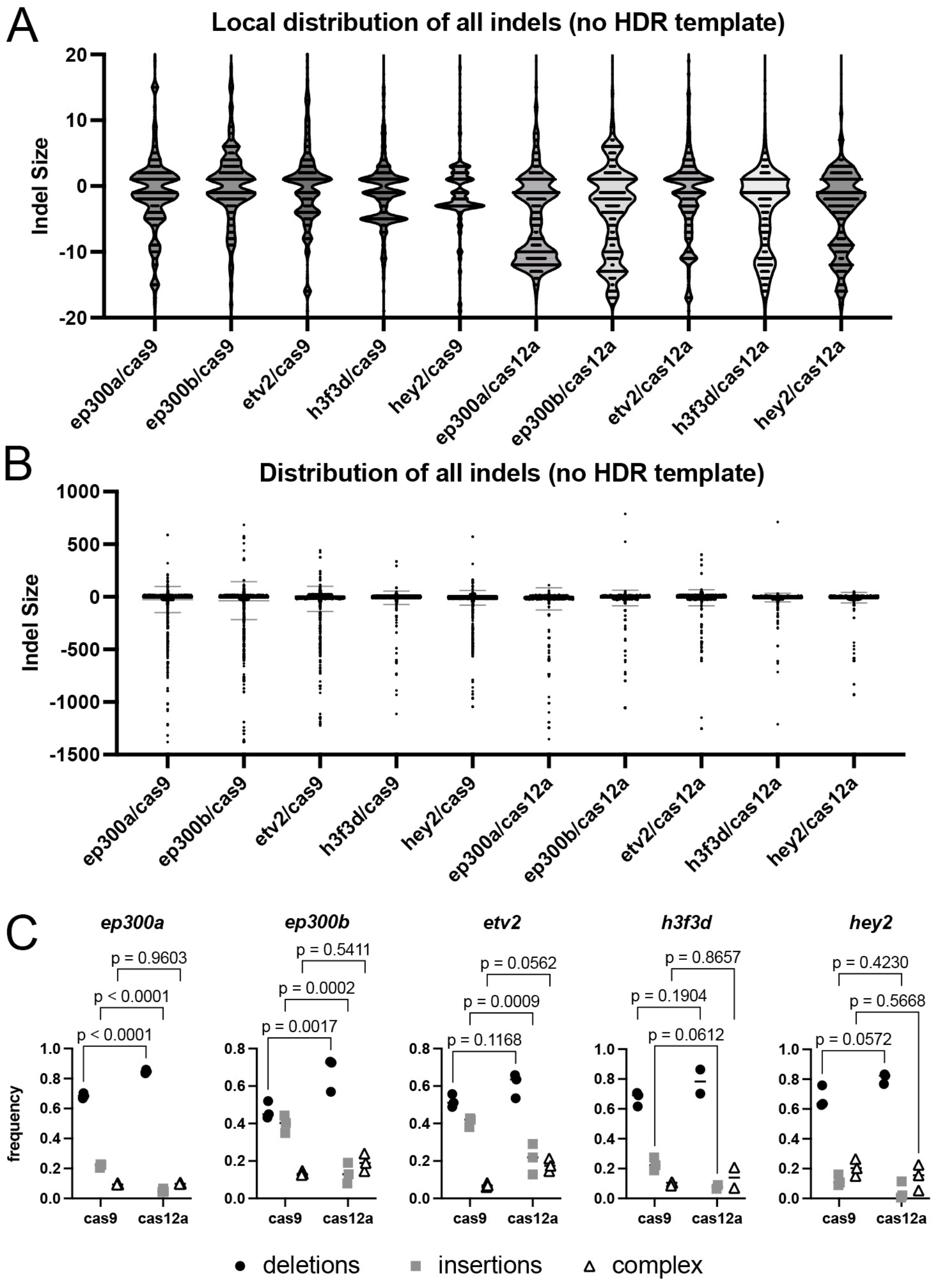

Fig. S3. Size distribution of nuclease-induced lesions. **A, B.** Distribution of insertion and deletion sizes (indels), indicated by positive and negative integers, respectively, at each target with indicated CRISPR nuclease on the X-axis. **A.** Range includes all indels. **B.** Subset of data in A. showing only "local" indels of 20 bp or less. **C.** Frequency of indicated lesion at each locus with indicated nuclease. 2-way ANOVA, Sidak's multiple comparison test. p values are indicated. Raw numbers are shown in **Table S4**.

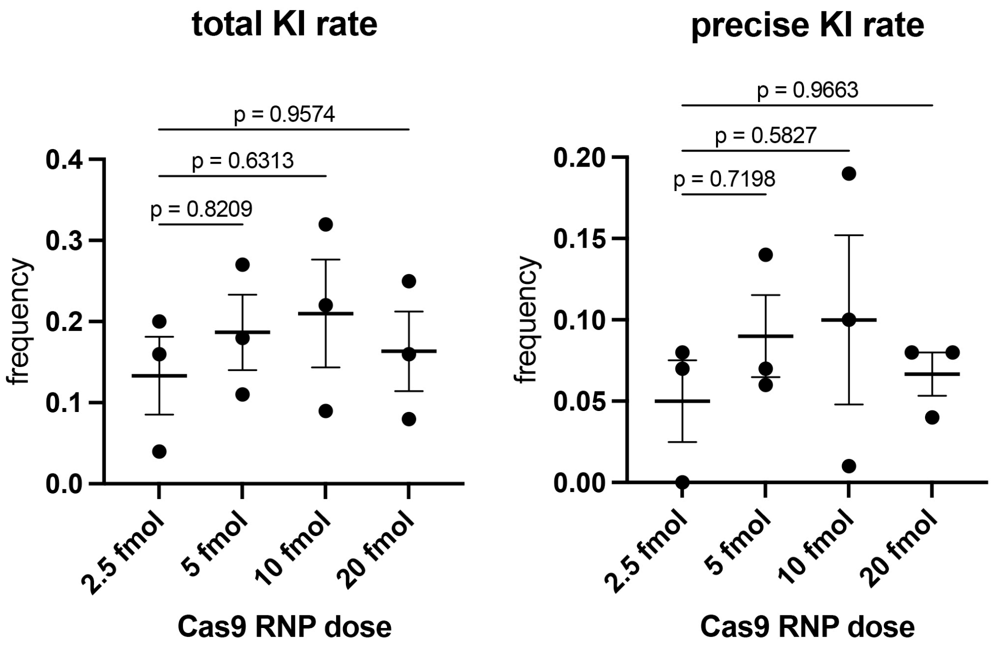

Fig. S4. **Cas9 RNP dose response at *h3f3d***. Total and precise knock-in (KI) rates at the *h3f3d* ATG with PCR-amplified 2'OMe-modified template and Cas9 RNP. The template in this case was amplified using gene-specific homology-matched PCR primers. Template amounts were constant across all samples and Cas9 RNP dose per embryo is indicated. 1-way ANOVA, Dunnett's multiple comparison; p-values are indicated.

Development • Supplementary information

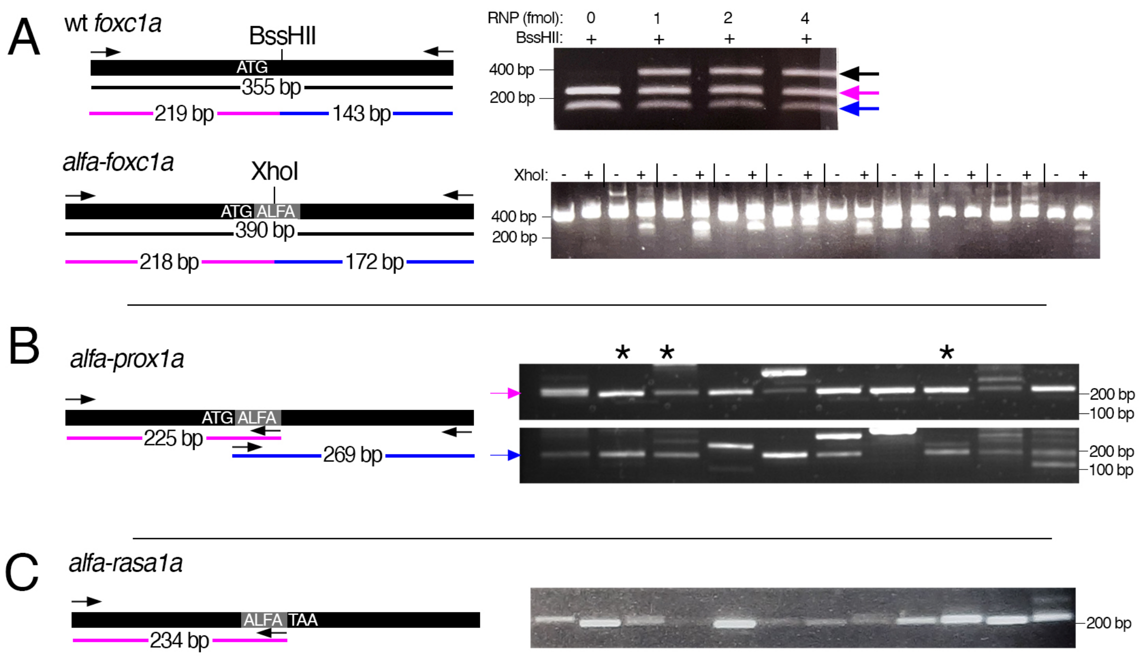

Fig. S5. Evidence of F0 knock-in at *foxc1a*, *prox1a*, and *rasa1a*. **A**. *Top left*, expected PCR and digest products from *foxc1a* wild type and precise insertion allele. *Top right*, gel showing PCR products digested with BssHII from pooled 24 hpf embryos. *Bottom right*, gel showing undigested and XhoI-digested PCR from 24 hpf individual embryos co-injected with *foxc1a* ATG Cas9 RNP and 2'-OMe-amplified PCR product. **B**. *Left*, expected PCR products from ALFA knock-in at the *prox1a* locus using anchored primers. *Right*, gel showing PCR products from anchored PCR flanking 5' (magenta) and 3' (blue) junction sites. Asterisk denotes individual embryos with correct size fragments at both junction sites. **C.** *Left*, expected PCR product from ALFA knock-in at *rasa1a* using an anchored primer to amplify across the 5' junction site. *Right*, gel showing PCR product from anchored PCR.

Development • Supplementary information

Table S1. Description of targets, CRISPR doses and associated information.

Available for download at
https://journals.biologists.com/dev/article-lookup/doi/10.1242/dev.204571#supplementary-data

Table S2. Raw viability and toxicity data.

Available for download at
https://journals.biologists.com/dev/article-lookup/doi/10.1242/dev.204571#supplementary-data

Table S3. Long-read sequencing-based quantification of repair events comparing CRISPR nuclease and HDR templates.

Available for download at
https://journals.biologists.com/dev/article-lookup/doi/10.1242/dev.204571#supplementary-data

Table S4. Distribution of indels in the absence of HDR template.

Available for download at
https://journals.biologists.com/dev/article-lookup/doi/10.1242/dev.204571#supplementary-data

Table S5. Long-read sequencing-based quantification of repair events at the *h3f3d* locus.

Available for download at
https://journals.biologists.com/dev/article-lookup/doi/10.1242/dev.204571#supplementary-data

Table S6. Oligonucleotide sequences used in this study.

Available for download at
https://journals.biologists.com/dev/article-lookup/doi/10.1242/dev.204571#supplementary-data

SUPPLEMENTARY MATERIALS AND METHODS

A general outline for design and implementation of targeted knock-in in zebrafish

1. Identify CRISPR targets at desired insertion site.

    - Site of the double-stranded break (DSB) should be fewer than 5 bp from the transgene insertion site.

    - Multiple CRISPR nucleases with different PAMs are now commercially available and all are active in zebrafish. If necessary, we recommend using alternatives to Cas9 to minimize the DSB/tag insertion distance:

        - Cas9, 5'-spacer-NGG-3', NEB and IDT
        - Sau Cas9, 5'-spacer-NNGRRT-3', NEB
        - Seq Cas9, 5'-spacer-NAGA-3', NEB
        - Cas12a, 5'-TTTN-spacer-3', NEB and IDT

    - Our current preference is to use a Cas9 or variants thereof, rather than Cas12a, since HDR-mediated insertion at the DSB will separate the PAM and spacer sequences thereby prevent recutting by the CRISPR. For Cas12a, you usually need to introduce base pair changes in the PAM.

    - We usually try to rely on restriction enzyme sites near the CRISPR-induced DSB for estimating nuclease activity. Therefore, we recommend checking for RE sites when identifying candidate CRISPR targets. The only limit we typically place on RE searches is that the enzyme be commercially available. Note that this feature is less of a priority than DSB/insert distance.

2. Test CRISPR activity

    - Start with tests in the range of 2 to 10 fmol per embryo
        - Most CRISPR RNPs in our hands show optimal activity in this range
        - modify range on follow-up tests as necessary

    - We always use recombinant protein to make CRISPR ribonucleoprotein complexes and inject at 1-cell stage

    - Assess morphology at 24 hpf
        - adjust dosage accordingly to minimize toxicity
        - if targeting the start codon, keep in mind that you may observe a *bona fide* mutant phenotype if the CRISPR is highly active. In this case, modify dose to minimize phenotypic penetrance and optimize knock-in

    - harvest genomic DNA from 10-30 embryos
        - crude extracts from NaOH-mediated lysis can be used here

    - PCR amplify, digest with restriction enzyme, analyze by agarose gel electrophoresis
        - alternatives are Sanger sequencing followed by Inference of CRISPR Edits (ICE) analysis or short-read deep sequencing

Development • Supplementary information

3. HDR template
    - we suggest waiting to design the template until CRISPR activity is confirmed

    - place insert as close to DSB as possible, while allowing for a functional allele (e.g. in-frame epitope tag)

    - We prefer to include restriction enzyme sites in the inserted sequence to allow for optimizing template knock-in conditions, as well as founder identification and for subsequent genotyping

    - use 50 bp homology arms

    - options for generating HDR template:
        - clone and PCR-amplify using modified PCR primers
            - 2'OMe-modified primers can be purchased from IDT
            - we prefer blunt cloning into pJET using a gBlock (IDT) comprising the homology arms and desired insert

        - use AltR HDR template gBlocks; can be purchased from IDT
            - alternatively purchase AltR ssODNs from IDT and anneal; this may depend on the total sequence length
            - AltR HDR templates are sequence-validated
            - Anecdotally, our results with these have been less consistent than the 2'OMe-amplified templates

    - We recommend testing optimal template amounts for co-injection
        - co-inject optimal RNP dose with increasing amounts of template; usually from 2.5 to 10 or 20 pg.

        - use 10 individual embryos for PCR testing
            - isolation by NaOH-mediated lysis

        - Use anchored PCR, or flanking PCR followed by digest with restriction site that is unique to the inserted sequence
            - make sure that flanking primers are outside of the homology arm sequences
            - restriction digest-based approach may provide better estimate of knock-in efficiency

        - Ideal conditions should yield greater than 30% of the embryos showing evidence of knock-in

        - optional: shotgun clone and sequence knock-in fragments to confirm that some are precise
            - we have not routinely been doing this, but recommend for those trying this for the first time

4. use optimal conditions to generate P0 fish
    - co-inject optimal RNP and HDR template dose

Development • Supplementary information

- inject 1-cell stage embryos
    - as early as possible into the blastomere
    - avoid the yolk!

5. Identify P0 founders
    - harvest pooled embryos (~30) from individual P0 outcrosses to wild type

    - screen for evidence of knock-in using PCR
        - one approach is to screen using both forward and reverse primers anchored in the inserted sequence
        - alternatively, use flanking primers followed by digest with a restriction enzyme that will only cut the inserted sequence

    - shotgun clone fragments from putative insertion alleles
        - founders can transmit a mix of alleles, therefore it is essential to verify these in the F1 embryos
        - to definitively identify precise insertions, it is important to use flanking, NOT anchored, primers for this step
        - we typically pre-screen clones for evidence of knock-in followed by sequencing of those positive clones

6. F1 carriers
    - As noted above, P0 founders can transmit multiple alleles. Therefore, it is essential to definitively confirm that F1 heterozygous fish bear the correct insertion allele
        - we identify F1 fish by finclip and PCR-based genotyping
        - upon first identification of new F1 lines, we recommend shotgun cloning PCR products to confirm the correct allele/insertion
        - once confirmed, subsequent genotyping can be performed by flanking PCR and RE digest, or anchored PCR.
