# Supplementary material for: Identifying optimal conditions for precise knock-in of exogenous DNA into the zebrafish genome
Source: Development. 2025 Jun 19;152(12):dev204571. doi: 10.1242/dev.204571 (PMC12211562; doi:10.1242/dev.204571)
Supplement: Supplementary file 1 [file develop-152-204571_review_history.pdf]

Identifying optimal conditions for precise knock-in of exogenous DNA into the zebrafish genome

Sarah Oikemus, Kai Hu, Masahiro Shin, Feston Idrizi, Aliece Goodman-Khan, Amy Kolb, Krishna S. Ghanta, Jonathan Lee, Atish Wagh, Scot A. Wolfe, Lihua J. Zhu, Jonathan K. Watts and Nathan D. Lawson
DOI: 10.1242/dev.204571

Editor: Steve Wilson

Review timeline

Original submission:	1 December 2024
Editorial decision:	20 January 2025
First revision received:	31 March 2025
Editorial decision:	25 April 2025
Second revision received:	6 May 2025
Accepted:	14 May 2025

Original submission

First decision letter

MS ID#: dev.204571

MS TITLE: Identifying optimal conditions for precise knock-in of exogenous DNA into the zebrafish genome

AUTHORS: Sarah Oikemus; Kai Hu; Masahiro Shin; Feston Idrizi; Aliece Goodman-Khan; Amy Kolb; Krishna S. Ghanta; Atish Wagh; Jonathan Lee; Scot Wolfe; Lihua J. Zhu; Jonathan Watts; Nathan D. Lawson

Dear Nathan,

I have now received all the referees' reports on the above manuscript, and have reached a decision. The referees' comments are appended below, or you can access them online: please go to:

As you will see, the referees express considerable interest in your work, but have some significant criticisms and suggestions for improving your manuscript. Reviewers 1 and 2 both find the manuscript challenging to read and suggest that in revision, you should make the text more accessible (while retaining sufficient methodological detail). If you are able to revise the manuscript along the lines suggested, I will be happy receive a revised version of the manuscript. Please also note that Development will normally permit only one round of major revision. If it would be helpful, you are welcome to contact us to discuss your revision in greater detail. Please send us a point-by-point response indicating your plans for addressing the referees' comments, and we will look over this and provide further guidance.

Reviewer 1

Oikemus (or Lawson?) et al. worked on improving knock-in by homology-directed repair (HDR) in zebrafish by testing two main variables: the nuclease (Cas9 or Cas12a) and the template design. For the template, they tested templates released from a plasmid or PCR-amplified with or without

© 2025. Published by The Company of Biologists under the terms of the Creative Commons Attribution License (https://creativecommons.org/licenses/by/4.0/).

chemical modifications in the primers. Another original point of this work is the use of long-read sequencing (PacBio) to quantify the mutations.

I think I understood most of the work but I must say this took a substantial effort for a paper of this size. And I say this as someone who work on projects very close to this paper, anyone that is even slightly outside to this exact niche field would quickly get lost. As it is, the current paper can only be understood, and thus be useful to, a minuscule group of experienced (and very patient) zebrafish geneticists. This is a shame, as the results could in theory be useful to e.g. a PhD student in developmental biology or neuroscience that wants to create a new epitope-tagged line as part of their project. This criticism is especially relevant here as the authors submitted to a developmental biology journal, not a genetics or genome editing one, so the work should be decipherable by this audience. When improving the presentation, I would advise the authors to keep this imaginary PhD student in mind. I still leave with a broadly positive opinion of the science, but I unfortunately spent the majority of the time trying to decipher the paper rather than thinking about the science. I tried to be as exhaustive as possible below to help the authors re-work their paper. In addition, I would strongly recommend having one clear cartoon with the main strategies tested, where we see the template being produced before injection, or the plasmid co-injected with Cas9 or restriction enzyme and getting released in the cell, etc. This would probably make everything a lot clearer!

Two broad criticisms before a bunch of point-by-point comments:

1) An original point of the paper is the use of PacBio long-read sequencing. It is the second sentence of the Abstract so I am assuming the authors want to advertise this. Unfortunately, it is then very superficially explained/argued. We are only given a couple of very jargony arguments (e.g. "jackpot effects" of normal PCR) which do not really explain what the long-read sequencing really helps with in the context of this work. As for the results, Fig. S4A seems to be the only figure where the "long" of long-read sequencing is really exploited. To be charitable with the authors, I do know, among others, that the complete insertion (~ 190 nt) would be difficult to sequencing with an Illumina read, but I have worked with NGS long/short-read sequencing before, this will not be obvious to the majority of readers, especially in the field of zebrafish genetics where many use Sanger sequencing. For example, simply mentioning "to accurately quantify repair events" is not sufficient, it would be more convincing to explain a precise example of an event that would not be detected by short-read sequencing.

2) Probably this criticism could be up for (a long) debate, but I tend to have different opinions than the authors about the use of p-values. The authors add p-values to every plot, regardless of sample sizes. I would personally avoid ever reporting a p-value on a sample size of three. This is not to say that these comparisons are useless because the sample sizes are too low, but rather that I, as a reader, do not regard the p-value as a relevant argument in this situation. I give a couple of more precise examples below. As a more general comment, I would much prefer that the authors try to convince me *without* using a p-value as a sort of magic arbiter that I would automatically have to accept. To give a third precise example (on top of the two below), in Fig. 2B, condition "mod" is always better than the two others for all five loci tested. Each p-value does *not* take this into account (the null hypothesis concerns each locus taken individually), but surely this is the argument that matters. Whether the p-value is < 0.01 or < 0.001 on a sample size of three changes almost nothing to my level of confidence here. This connects to a broader criticism: I wish the authors would put more effort into actually interpreting their results, rather than just stating specific comparisons as significant or not. For example, the primers with phosphorothioate linkages did not help; any idea why? Does that tell us anything about what is going in the cell where the templates are injected?

Point-by-point comments:

- There seems to be an issue with the order of authors? Or it is just the way Development displays the order. The manuscript (by the authors) lists Oikemus as first / Lawson as last, but the first page of the draft I received (I supposed generated from a submission form) lists Lawson first / Oikemus third.

© 2025. Published by The Company of Biologists under the terms of the Creative Commons Attribution License (https://creativecommons.org/licenses/by/4.0/).

- The title should be more precise. There are so many papers working on improving knock-in in zebrafish, the authors will get more visibility if they emphasise what they think the readers should remember about their work (maybe Cas12 or long reads or chemically-modified PCR templates).

- There is some confusion across the paper (and across the field to be honest, the authors are not the only ones to blame) about DNA repair of double-strand breaks, specifically in zebrafish. My understanding is as follows. In zebrafish embryos, the great majority of double-strand breaks (at least those made by Cas9) are repaired by microhomology-mediated end-joining (MMEJ), or more precisely DNA polymerase Î¸-mediated end-joining (TMEJ). Not NHEJ. Thyme 2016 (10.1016/j.celrep.2016.03.072) shows this quite convincingly. Embryos deficient in PolÎ¸ die after injection with Cas9 because they cannot (at least not fast enough) repair double-strand breaks. Mutational signatures also show this, I believe the majority of indels tend to be deletions with microhomologies. Some breaks are probably repaired by NHEJ but it is really not the main repair zebrafish embryos use, while it is the only pathway mentioned here. Furthermore, I believe NHEJ tend to be quite accurate (not error-prone), while the authors interpret it as the sole source of indels. The authors should correct this. In my opinion, an appropriate and brief wording would be e.g. "repaired mostly by MMEJ".

- I liked the introduction paragraph on Cas12a. I have never looked into details how this nuclease works and this was nicely summarised.

- The introduction paragraph on long-read sequencing is lacking. The authors mention "size bias" of short-read sequencing but do not argue how their long-read approach is better. My understanding is that the size bias of short-read sequencing arises from the PCR. The approach here is still based on PCR, would it not suffer from the same bias? I think I can guess the answer because I vaguely understand how the PacBio method works but the authors should explain.

- "This insert was flanked by […] universal Cas9 gRNA sites", I am confused by "universal" here. Reading the sentence literally, it sounds like a site that every gRNA recognises, but that cannot be right.

- "For PCR-amplified template, […]", should state clearly in this sentence that they are amplified from the above-mentioned plasmids. Took me some time to understand but it is crucial.

- "primers complementary to the I-SceI sites"; is this approach really used in the field? Can the authors argue why? Although I never studied in great detail the HDR mechanism, I can guess that there is some annealing happening at some point between the homology arms and the genome, and therefore that you should ideally not have non-complementary sequences there. I do not know the cost of the modified primers (maybe that is the argument behind it?); but why would you not simply design the primers to anneal at the edge of the homology arms? Reading the Methods confuses me further… The authors first bought gBlocks (synthetic double-stranded DNA fragments) with the homology arms and the epitope tag. Why insert it into a plasmid only to release it with Cas9/restriction enzyme and purify it again? Is it to check single clones for synthesis errors? I thought at first it would be to compare vs. plasmid injection, but I do not think Fig. 2 has this condition? But the legend does not say what the conditions are, which does not help my work.

- "In cases where the insertion would not disrupt the CRISPR target in the homology arms"; I am quite confused. Can the authors try to re-word this? For both Cas9 and Cas12a, the cut is within the protospacer (CRISPR target) so it would disrupt it somewhat. Do the authors mean that the insertion would re-install the entire CRISPR target because it is present in the homology arm?

- Similar criticism for the part in the results "The use of PacBio…". "jackpot effects" is jargony/opaque, the average reader will not understand what is meant. I myself can only guess because I vaguely know how the PacBio sequencing works; I think the UMIs keep track of unique genomes?

- About 2'OMe primers: do I understand correctly these only appear later in the manuscript? It would be clearer to introduce them when they start being relevant.

© 2025. Published by The Company of Biologists under the terms of the Creative Commons Attribution License (https://creativecommons.org/licenses/by/4.0/).

- "Most Cas9 indels were under 10 bp […]", the reader is sent to Fig. S4A but really the text does not talk about this plot until only six lines later if I understand correctly ("also induced much larger deletions"). I found this a bit confusing. Could swap the two plots for example.

- "from plasmid-release templates only at […]", the "only" makes this sentence impossible to understand for me. There is robust nuclease activities (start of the sentence), OK, so we expect high KI, but "very low total knock-in rates" only at h3f3d and hey2, so that should mean the other three are good? They are not though (Fig. 2B). Do the authors mean "detectable"? Like "knock-in was only detectable at h3f3d and hey2".

- The authors separate total / precise KI rate but never quite define those classes in the main text, only in the Methods. The authors should explain the first time what the mean by total/precise and show a couple of example reads in a figure (e.g. four reads: reference, precise KI, 5'-indel, 3'-indel). Again, I can follow, but a PhD student in developmental biology will not.

- "Variable incorporation of PAM mutations": I cannot understand the link with the rest of the sentence "suggesting that homology distances shorter than […]" (our imaginary PhD student definitely will not either!). Also, can the authors suggest how those events (incomplete insertion of the template, if I understand correctly) occur?

- "Levels of nuclease activity […]" and Fig. 2D,E: in my opinion this is a good example of over-reliance on p-values. From mechanistic understanding of DNA repair in general and HDR in particular, we know almost for certain that a break is required for knock-in to occur at the locus; is that fair? So the non-significant p-value here is likely a false negative, i.e. there simply *must* be a correlation between nuclease activity and KI rates and the p-value would probably be significant if there were more samples/more loci. So why report and discuss this result if we think it is unreliable? Here statistical significance does not guarantee that the result is significant in the sense that the readers should pay attention to it. I would personally not report this result in my own work as I believe it is highly likely to be a misleading false negative. In fact, the authors probably agree with me here as the next sentence states "Similarly, […] a positive, but insignificant, correlation between KI rates and RNP dose" (i.e. this positive correlation is interpreted even though not-significant, but the previous one was interpreted as not correlating, double standards!).

- I have a similar criticism (but reversed) for "precise KI rates significantly and inversely correlated with distance" and Fig. 2F: sure, the p-value is below 0.05, but are we really convinced given the number of datapoints and the distribution of the data? I would certainly not put my own belief in this result at "97% chance that there is a linear relation between distance and KI rate". You can get a p-value at ~ 0 (< $2.2 \times 10{-}16$, the smallest number allowed in R) with just three datapoints; that does not necessarily mean it is convincing. Also, we are testing for a linear relation but half the datapoints are 0 and there is clearly no relation distance vs. KI rate in the non-null datapoints, so just logically there is no linear relation detected here. The 7 and 5 bp distances (i.e. almost equal) are at the exact two extremes of the distribution (null KI rate and maximum KI rate), which is a clear counter-example to this putative linear relation. Again, in this case, the fact that the p-value is significant would give me a little bit of confidence but probably not enough to report such a plot/result in my own work. The way to convince me that this relationship is real is not to impress me with a low p-value but rather with a logical argument based on putative mechanisms of what might be happening during HDR.

- "distance between homology start […] (Fig. 1A-C)", I think it should be Fig. 1F?

- "We find that chemically modified PCR templates significantly outperformed in vivo-released fragments". I thought for a while that the authors injected whole plasmids, which were somehow cut within the cell before HDR (e.g. "in vivo released"), but I could not understand how this works in practice, especially with the restriction enzyme. Now, looking at the Methods, it seems like the HDR template is released from the plasmid *in vitro* prior to injection? EDIT: digging more into the Methods, I think my first interpretation was correct. Again, is the co-injection of a whole plasmid with a restriction enzyme/Cas9 RNP really used in the field? Why? I am especially puzzled by the restriction enzyme; would it not wreak havoc in the genome? I see the recognition sequence is fairly long, but also that some degeneracy is tolerated… On my initial confusion: the authors should

© 2025. Published by The Company of Biologists under the terms of the Creative Commons Attribution License (https://creativecommons.org/licenses/by/4.0/).

be much clearer in the results (not only in the methods) that e.g. a restriction enzyme is co-injected.

- About "In mammalian cells, the 3'-OH groups of single-stranded DNA donors [...]" and after: I find it very difficult to follow the authors here; I assume the majority of readers would also get lost. Unfortunately, this makes it hard to judge the results as I'm not quite sure what is being asked. To point the authors to the issues:
-- "need to be unblocked": expects too much prior knowledge from the reader, I myself do not know exactly what is meant (i.e. what would "blocked" look like?).
-- Same for "3'-terminus of the donor might be extended [...]. Thus, non-homologous nucleotides [...]"; this logic will be impossible to understand for the majority of readers. I need to do a lot of guess work to complete the gaps. My current guess: the homology arm anneals to the complementary genomic strand and is polymerised by an endogenous DNA polymerase using the genome as template. So, if you have mismatches in the homology arm, it is like having mismatches in a PCR primer, this extension process will not work as well and HDR may be affected. Is this the logic? It is interesting but not trivial to understand, it requires much better explanation to be communicated, especially as the reader needs this knowledge to interpret the following results.
-- Took me multiple re-reads and a long stare at Fig. 1 to understand (I think) what are those "non-homologous nucleotides". I first thought the authors meant the A>G edit to inactivate the PAM of h3f3d? But then why plural "at these positions"... Now I think the authors mean the entire I-SceI site + the few nucleotides between the homology arm and the I-SceI site? Even if this is the correct interpretation, it requires much better explanation as it asks too much effort from the reader.
-- "2'OMe modified primers": I understand this modification was mentioned before, but I doubt most readers will still have it in mind here. Perhaps a better wording would be like "synthesized primers.... As before, these included 2'-OMe modifications to reduce concatemerization".
-- "universal PCR-amplified template": I have no idea what "universal" means here.
-- "bearing non-homologous residues"; residue in my mind means amino acid? Correction: I see that it can be used for a nucleotide! I have never heard it used to say something else than amino acid though, so I would not use it to avoid confusing the reader, especially as "non-homologous nucleotide" is used just before.
-- "Since previous studies [...]": are these the same v1 / v2 primers as introduced at the start ("Only v1 primers [...]"), then I would not introduce them so early, only here.
-- "phosphorothioate linkages": need some explanation. What are they; why are they used here (I know they were mentioned before, but without any explanation either). I know what they are, and I think I can guess why they could be useful, but again, think about the PhD student in developmental biology!

- Given the introduction sentences of this paragraph, I assume that the authors interpret the "five-fold increase in precise knock-in rate" as confirmation that the template has to be extended by this unknown endogenous polymerase? This hypothesis is raised (very superficially) but then the interpretation does not circle back to it.

- Fig. 1A legend "Line located above or below sequence indicates strand, respectively", I do understand what this line! There is a sort of ruler in the middle of the sequences but no arrows or anything so that tells me the Forward or Reverse genomic directions. Also, "respectively" is used to match two things to two others (here the sentence says above & below both mean strand, so why respectively).

- Fig. 1A: I would annotate the PAMs (e.g. bold is often used, or a frame). It will also make it easier to understand the PAM mutations (*).

- Fig. 2A legend: "hey2 where a Mann-Whitney test was used"; I guess this is not very important as the p-value is non-significant anyways, but I found this a little surprising. If the authors think that the KI rates tend to follow a normal distribution (probably a reasonable assumption), why not use t-tests for all?

- Fig. 2: the legend does not say what is "sce", "uni", "mod". If I was a simple reader, I would probably have given up trying to understand the paper at this stage!

© 2025. Published by The Company of Biologists under the terms of the Creative Commons Attribution License (https://creativecommons.org/licenses/by/4.0/).

- Fig. S1: legend does not explain the abbreviations "undig", "uninj". Again, I can guess, but the authors need to think about where they want to publish / who their target audience is.

- Fig. S3: the cartoon is OK but the legend is very bare-bone, one certainly cannot understand how the method works (e.g. what the UMIs are for) from this alone.

- Fig. S5: I imagine that frequency is "out of all epitope insertions" or something like this? Is it not mentioned.

Some minor typos, wording, etc:

- Long/short-read sequencing with hyphen or not is not consistent (e.g. both "short-read" and "long read" in the abstract). I think the adjective should have a hyphen (e.g. long-read sequencing), but not if used as a noun ("long reads").

- Consistency of tenses (e.g. "we find…" and "we found…" in abstract).

- Consistency about double strand(ed) breaks / double-strand breaks.

- "For PCR-amplified template, we used […]", I think *templates?

- "We compared several different HDR templates", can delete "several".

- "nuclease activity by calculating insertion or deletion (indel)", the abbreviation "indel" was already introduced just above.

<u>Reviewer 2</u>

SUMMARY OF THE ADVANCE MADE IN THIS PAPER AND ITS POTENTIAL SIGNIFICANCE TO THE FIELD

The ability to introduce precise targeted insertions using CRISPR-based genome editing has facilitated phenotypic evaluation of specific mutations in human cells and animal models, like the zebrafish. In the manuscript by Oikemus et al., variations of CRISPR-based exogenous sequence knock-in into the zebrafish are presented with the goal of providing the key features of an optimized strategy. While technical advances and optimization of this approach are of tremendous interest in the field, the current report lacks sufficient detail and validation on which to conclusively "hang one's hat". In particular, as a methods paper, some key technical details require expansion and demonstration of the applicability of this approach incorporating epitope tags for real biological experiments would be highly beneficial.

SUGGESTIONS TO AUTHORS
Major issues:

1. Introduction : The concept of MMEJ is not discussed in this section, although multiple methods mentioned here utilize it to generate the knock-ins.

2. What is the significance of the 2'-O-Methyl-modified (2'OMe) ribose bases? Their predicted activity should be explained.

3. The significance and utility of the v1 vs. v2 primers should also be explained.

4. The manuscript is somewhat confusing to follow regarding the various different constructs being generated and tested. Understanding would be greatly improved by including graphical summaries/schematics in each figure prior to the data in which that strategy is employed.

© 2025. Published by The Company of Biologists under the terms of the Creative Commons Attribution License (https://creativecommons.org/licenses/by/4.0/).

5. The legend for Figure 2 is insufficiently detailed regarding the specific conditions tested. Moreover, the text related to Figure 2 is also not completely consistent with what is displayed in the figure, and again insufficient detail is provided. For example, the phrase "only at h3f3d and hey2" does not explain what occurred for the other genes in question and which nuclease is being employed. The data (text, figure and figure legend) need to be revised for completeness and clarity.

6. Total vs. precise knock ins should be defined.

7. The universal vs. gene-specific nature of the modified primers was not introduced in the text. It is not clear from the manuscript what roles these RNA-based oligo parts play since they are separated by the I-SceI sites from the homology arms.

8. Why did the authors only evaluate h3f3d with universal vs. gene-specific primers? It is unclear why all 4 combinations of universal vs homology-matched and v1 vs. v2 design versions were not tested. Why was there selected injection of data from one additional design combination but only in the germline transmission table? Additional genes should be tested to bolster the authors' conclusions around optimal conditions.

9. The authors state they did not observe any differences comparing templates amplified with v1 or v2 primers (Fig S7), however, there was a significant decrease in the 5' tag deletion rate for the v2 primers. Please address and provide further details.

10. It would be highly advantageous to demonstrate the typical application of an epitope tagged line in terms of immunofluorescence and/or Western blotting. This would strengthen the practical utility of the method from the conceptual design to the direct biological application.

Minor issues:

1. Introduction: Although technically in Medaka, a paper in eLife (https://elifesciences.org/articles/75050v2) provides an example where biotin-mSA interaction acts as the stimulatory factor for knock-in. It may be worth mentioning it here as well.

2. Results and Discussion: Please be more precise than "approximately 50 bp homology arms"? Was it always 50 bp and what were the reasons to choose other lengths?

3. Results and Discussion: The phrase "nearly complete loss of a restriction site" is not accurate due to heteroduplexes being resistant to digestion. The authors should modify to say something like "inactivation of digestion at an adjacent restriction site".

4. Results and Discussion: It is not clear exactly how and when the plasmid templates were cut to prepare the templates. It would be helpful to explain this at the first mention of "Plasmid-released" templates.

5. Results and Discussion: The phrase "note that dose-response results were obtained using homology matched HDR templates, see below" implies that the relevant information was not introduced. The authors should edit the text to make such references unnecessary.

© 2025. Published by The Company of Biologists under the terms of the Creative Commons Attribution License (https://creativecommons.org/licenses/by/4.0/).

6. Results and Discussion: Table 1 and associated text -please include details about the nuclease used to improve readability.

7. Results and Discussion: Figure S2: Please describe abbreviations used across the x-axis in the figure legend.

8. Materials and Methods: The sentences "For templates released from plasmids, 50pg/µl of plasmid DNA was mixed with either 1 unit/µl I-SceI enzyme or 5µM Cas9 universal RNP and injected at 10 pg template DNA per embryo. For PCR-amplified templates, we injected 2.5 pg per embryo and all injection volumes were 2 nl." is not internally consistent. Plasmid DNA has incorrect units and its volume is not indicated. PCR template injection mix is formulated in a different way than the plasmid DNA. Please formulate them the same way.

Reviewer 3

Reviewer comments for the authors:

In this manuscript, Oikemus et al. present conditions for improving precise insertion of exogenous DNA into the zebrafish genome. The authors applied quantitative long read sequencing to identify optimal conditions for precise targeted insertion in zebrafish. In this manuscript, they report that 1) chemically modified templates out-perform those released in vivo from a plasmid, 2) Cas9 and Cas12a nucleases performed similarly for targeted insertions, 3) precise editing rates were dependent on the distance between a double-stranded break and the start of homology, and 4) inclusion of non-homologous base pairs in homology templates drastically reduced precise editing rates.

By using the presented optimized parameters, Oikemus et al. achieved germline founder rates of greater than twenty percent for precise insertions.

The presented research is technically sound and contains appropriate standardized controls.

The manuscript here presents an additional resource for gene editing strategies, in particular knockin strategies, warranting the future use of their protocol for the research community.

Only the following minor points were identified that would need further clarification:

- Introduction: "An important variable across targeted knock-in studies is the HDR template." This again comes up in the results part. It is unclear why the authors refer to HDR template and CRISPR nuclease as variables. Suggestion to rephrase "variable" with "component" or similar.

- Suggestion to shorten the introduction to make it more concise for readers.

- "All subsequent injections were performed by three different lab members to generate triplicate data points and mimic real-world application." Great standardized practice! Thorough work!

- Could the authors include a comment on how affordable PacBio sequencing is at the moment for the readers?

- "Since previous studies using chemically modified templates had incorporated phosphorothioate linkages," Please provide reference.

- Refer to knockin earlier in the introduction.

- Clarify knockin template better in the results part of Fig. 1A.

- Methods and results part: please specify whether it was injected into the yolk or cell.

© 2025. Published by The Company of Biologists under the terms of the Creative Commons Attribution License (https://creativecommons.org/licenses/by/4.0/).

- Please provide a sentence in the text on why epitope tags were only inserted 5' of the target genes.

- Clarify if tag is inserted in frame.

- Please clarify "jackpot effects" for the broader readership in the results part.

- We enjoyed reading the word monsters! But please clarify Monsters in FigS. 2. Please leave in Monsters but specify the meaning better and give examples of phenotypes describing monsters.

- Suggestion to include for bullet point takeaways or graphical abstract.

First revision

Author response to reviewers' comments

We thank the reviewers for their time and effort in reviewing our manuscript. To accommodate the suggested changes, we have switched the format from a Report to an Article, with permission from the handling editor. Given the extensive revisions, we have not provided a document with the tracked changes.

Comments from the Reviewers:

Reviewer 1
1) An original point of the paper is the use of PacBio long-read sequencing. It is the second sentence of the Abstract so I am assuming the authors want to advertise this. Unfortunately, it is then very superficially explained/argued. We are only given a couple of very jargony arguments (e.g. "jackpot effects" of normal PCR) which do not really explain what the long-read sequencing really helps with in the context of this work. As for the results, Fig. S4A seems to be the only figure where the "long" of long-read sequencing is really exploited. To be charitable with the authors, I do know, among others, that the complete insertion (~ 190 nt) would be difficult to sequencing with an Illumina read, but I have worked with NGS long/short-read sequencing before, this will not be obvious to the majority of readers, especially in the field of zebrafish genetics where many use Sanger sequencing. For example, simply mentioning "to accurately quantify repair events" is not sufficient, it would be more convincing to explain a precise example of an event that would not be detected by short-read sequencing.
We provide a more expansive overview of long- versus short-read sequencing. We also provide additional explanation of “jackpot” effects, although this has to do with PCR and the rationale for using the unique molecular identifiers, rather than whether one is using long- or short-read sequencing. In any case, both points are addressed in the revised manuscript.

*2) Probably this criticism could be up for (a long) debate, but I tend to have different opinions than the authors about the use of p-values. The authors add p-values to every plot, regardless of sample sizes. I would personally avoid ever reporting a p-value on a sample size of three. This is not to say that these comparisons are useless because the sample sizes are too low, but rather that I, as a reader, do not regard the p-value as a relevant argument in this situation. I give a couple of more precise examples below. As a more general comment, I would much prefer that the authors try to convince me *without* using a p-value as a sort of magic arbiter that I would automatically have to accept. To give a third precise example (on top of the two below), in Fig. 2B, condition "mod" is always better than the two others for all five loci tested. Each p-value does *not* take this into account (the null hypothesis concerns each locus taken individually), but surely this is the argument that matters. Whether the p-value is < 0.01 or < 0.001 on a sample size of three changes almost nothing to my level of confidence here. This connects to a broader criticism: I wish the authors would put more effort into actually interpreting their results, rather than just stating specific comparisons as significant or not. For example, the primers with phosphorothioate linkages did not help; any idea why? Does that tell us anything about what is going in the cell where the templates are injected?*

© 2025. Published by The Company of Biologists under the terms of the Creative Commons Attribution License (https://creativecommons.org/licenses/by/4.0/).

We applied standard statistical analyses that are typically required at any journal for publication. The reviewer can interpret the p-values as they see fit. Given the incomplete dataset regarding the V1 and V2 modified primers, the V2 data have been removed from the revised manuscript. In some cases, we have expanded on possible mechanistic explanations of observed results, in others we have not.

Point-by-point comments:
- There seems to be an issue with the order of authors? Or it is just the way Development displays the order. The manuscript (by the authors) lists Oikemus as first / Lawson as last, but the first page of the draft I received (I supposed generated from a submission form) lists Lawson first / Oikemus third.
The correct author order is on the manuscript itself. I do not otherwise know how the Development manuscript submission system handles this.

- The title should be more precise. There are so many papers working on improving knock-in in zebrafish, the authors will get more visibility if they emphasise what they think the readers should remember about their work (maybe Cas12 or long reads or chemically-modified PCR templates).
The title has been changed in the revised manuscript.

- There is some confusion across the paper (and across the field to be honest, the authors are not the only ones to blame) about DNA repair of double-strand breaks, specifically in zebrafish. My understanding is as follows. In zebrafish embryos, the great majority of double-strand breaks (at least those made by Cas9) are repaired by microhomology-mediated end-joining (MMEJ), or more precisely DNA polymerase θ-mediated end-joining (TMEJ). Not NHEJ. Thyme 2016 (10.1016/j.celrep.2016.03.072) shows this quite convincingly. Embryos deficient in Polθ die after injection with Cas9 because they cannot (at least not fast enough) repair double-strand breaks. Mutational signatures also show this, I believe the majority of indels tend to be deletions with microhomologies. Some breaks are probably repaired by NHEJ but it is really not the main repair zebrafish embryos use, while it is the only pathway mentioned here. Furthermore, I believe NHEJ tend to be quite accurate (not error-prone), while the authors interpret it as the sole source of indels. The authors should correct this. In my opinion, an appropriate and brief wording would be e.g. "repaired mostly by MMEJ".
This has been addressed in the revised manuscript.

- The introduction paragraph on long-read sequencing is lacking. The authors mention "size bias" of short-read sequencing but do not argue how their long-read approach is better. My understanding is that the size bias of short-read sequencing arises from the PCR. The approach here is still based on PCR, would it not suffer from the same bias? I think I can guess the answer because I vaguely understand how the PacBio method works but the authors should explain.
Multiple steps in preparing a short-read sequencing library for the Illumina platform will bias against longer fragments. This includes PCR, bead-based purification, and flow-cell clustering. These are now more clearly listed with appropriate literature citations in the revised manuscript. We do not provide a detailed description of PacBio except to mention that it a single molecule-based approach that allows reads lengths more than 10kb, which is what is relevant for the reader to know here. Those interested in the finer details of PacBio sequencing can look up the citation included in the text.

- "This insert was flanked by […] universal Cas9 gRNA sites", I am confused by "universal" here. Reading the sentence literally, it sounds like a site that every gRNA recognises, but that cannot be right.
This refers to a vector-targeted spacer sequence described by Wierson et al. in their “Geneweld” approach where it was called a “universal guide RNA (UgRNA)”. This is now more clearly defined and described in the revised manuscript.

- "For PCR-amplified template, […]", should state clearly in this sentence that they are amplified from the above-mentioned plasmids. Took me some time to understand but it is crucial.
This is made clearer in the revised manuscript.

© 2025. Published by The Company of Biologists under the terms of the Creative Commons Attribution License (https://creativecommons.org/licenses/by/4.0/).

- "primers complementary to the I-SceI sites"; is this approach really used in the field? Can the authors argue why? Although I never studied in great detail the HDR mechanism, I can guess that there is some annealing happening at some point between the homology arms and the genome, and therefore that you should ideally not have non-complementary sequences there. I do not know the cost of the modified primers (maybe that is the argument behind it?); but why would you not simply design the primers to anneal at the edge of the homology arms? Reading the Methods confuses me further… The authors first bought gBlocks (synthetic double-stranded DNA fragments) with the homology arms and the epitope tag. Why insert it into a plasmid only to release it with Cas9/restriction enzyme and purify it again? Is it to check single clones for synthesis errors? I thought at first it would be to compare vs. plasmid injection, but I do not think Fig. 2 has this condition? But the legend does not say what the conditions are, which does not help my work.

As described in the introduction, linear template released from a plasmid is one of the common HDR templates used in the literature; we refer the reviewer to the numerous citations in the introduction on this point. Alternatively, PCR-amplified templates have been used. Our goal was to compare them side-by-side; this is more explicitly stated in the revised manuscript. Since nuclease-mediated plasmid release results in non-homologous nucleotides on the HDR template, we purposely avoided using perfectly homologous PCR template so that they would be similar in this regard to the plasmid-released templates. If the PCR templates had been homology-matched in the first set of experiments, we would not have been able to discern effects from the template type (plasmid-released or 2'OMe-PCR-amplified) versus differences due to homology-matched arms. The rationale for using non-homologous base pairs on the PCR-amplified HDR template is more clearly presented in the revised manuscript.

- "In cases where the insertion would not disrupt the CRISPR target in the homology arms"; I am quite confused. Can the authors try to re-word this? For both Cas9 and Cas12a, the cut is within the protospacer (CRISPR target) so it would disrupt it somewhat. Do the authors mean that the insertion would re-install the entire CRISPR target because it is present in the homology arm?

As described in the introduction, the orientation of the PAM and spacer for Cas12a (5'TTTN-spacer-3') is opposite of that for Cas9 (5'-spacer-NGG-3'). The spacer for Cas12a can also be longer and the cut site occurs quite distal to the PAM. Consequently, an indel or even large insertion at a cut site introduced by Cas12a may leave much of the spacer and PAM still intact allowing for recutting. This is different from Cas9 where the cut site is very close to the PAM and any insertion will separate PAM and spacer, preventing recutting. This is now stated more clearly in the revised manuscript.

- Similar criticism for the part in the results "The use of PacBio…". "jackpot effects" is jargony/opaque, the average reader will not understand what is meant. I myself can only guess because I vaguely know how the PacBio sequencing works; I think the UMIs keep track of unique genomes?

A more comprehensive and clearer explanation of these points is provided in the revised manuscript.

- About 2'OMe primers: do I understand correctly these only appear later in the manuscript? It would be clearer to introduce them when they start being relevant.

They were used in the initial comparisons and were described when introduced. We provide additional information on their use in the revised manuscript.

- "Most Cas9 indels were under 10 bp […]", the reader is sent to Fig. S4A but really the text does not talk about this plot until only six lines later if I understand correctly ("also induced much larger deletions"). I found this a bit confusing. Could swap the two plots for example.

This has been changed in the revised manuscript.

- "from plasmid-release templates only at […]", the "only" makes this sentence impossible to understand for me. There is robust nuclease activities (start of the sentence), OK, so we expect high KI, but "very low total knock-in rates" only at h3f3d and hey2, so that should mean the other three are good? They are not though (Fig. 2B). Do the authors mean "detectable"? Like "knock-in was only detectable at h3f3d and hey2".

KI rates are now more explicitly described in the revised manuscript.

© 2025. Published by The Company of Biologists under the terms of the Creative Commons Attribution License (https://creativecommons.org/licenses/by/4.0/).

- The authors separate total / precise KI rate but never quite define those classes in the main text, only in the Methods. The authors should explain the first time what the mean by total/precise and show a couple of example reads in a figure (e.g. four reads: reference, precise KI, 5'-indel, 3'-indel). Again, I can follow, but a PhD student in developmental biology will not.

These are more clearly defined in the revised manuscript.

- "Variable incorporation of PAM mutations": I cannot understand the link with the rest of the sentence "suggesting that homology distances shorter than […]" (our imaginary PhD student definitely will not either!). Also, can the authors suggest how those events (incomplete insertion of the template, if I understand correctly) occur?

We have removed the data and associated figure regarding PAM conversion rates from the revised manuscript. These data were incomplete and did not contribute to pertinent points regarding knock-in optimization.

*- "Levels of nuclease activity […]" and Fig. 2D,E: in my opinion this is a good example of over-reliance on p-values. From mechanistic understanding of DNA repair in general and HDR in particular, we know almost for certain that a break is required for knock-in to occur at the locus; is that fair? So the non-significant p-value here is likely a false negative, i.e. there simply *must* be a correlation between nuclease activity and KI rates and the p-value would probably be significant if there were more samples/more loci. So why report and discuss this result if we think it is unreliable? Here statistical significance does not guarantee that the result is significant in the sense that the readers should pay attention to it. I would personally not report this result in my own work as I believe it is highly likely to be a misleading false negative. In fact, the authors probably agree with me here as the next sentence states "Similarly, […] a positive, but insignificant, correlation between KI rates and RNP dose" (i.e. this positive correlation is interpreted even though not-significant, but the previous one was interpreted as not correlating, double standards!).*

It's not clear what the reviewer wants us to change here.

- I have a similar criticism (but reversed) for "precise KI rates significantly and inversely correlated with distance" and Fig. 2F: sure, the p-value is below 0.05, but are we really convinced given the number of datapoints and the distribution of the data? I would certainly not put my own belief in this result at "97% chance that there is a linear relation between distance and KI rate". You can get a p-value at ~ 0 ($< 2.2 \times 10-16$, the smallest number allowed in R) with just three datapoints; that does not necessarily mean it is convincing. Also, we are testing for a linear relation but half the datapoints are 0 and there is clearly no relation distance vs. KI rate in the non-null datapoints, so just logically there is no linear relation detected here. The 7 and 5 bp distances (i.e. almost equal) are at the exact two extremes of the distribution (null KI rate and maximum KI rate), which is a clear counter-example to this putative linear relation. Again, in this case, the fact that the p-value is significant would give me a little bit of confidence but probably not enough to report such a plot/result in my own work. The way to convince me that this relationship is real is not to impress me with a low p-value but rather with a logical argument based on putative mechanisms of what might be happening during HDR.

The DSB/insert distance effect has been observed in several previous studies, two of which are cited here, as well as numerous other studies, including those prior to the advent of CRISPR nuclease (e.g. using TALE nucleases). Previous work has only speculated about potential mechanisms regarding the nature of this effect, with the central take-home messages being: it is important to minimize this distance. We see the same here and have chosen not to speculate on the mechanism.

- "distance between homology start […] (Fig. 1A-C)", I think it should be Fig. 1F?

The figure numbers have changed in the revised manuscript. This was corrected in the process.

*- "We find that chemically modified PCR templates significantly outperformed in vivo-released fragments". I thought for a while that the authors injected whole plasmids, which were somehow cut within the cell before HDR (e.g. "in vivo released"), but I could not understand how this works in practice, especially with the restriction enzyme. Now, looking at the Methods, it seems like the HDR template is released from the plasmid *in vitro* prior to injection? EDIT: digging more into the Methods, I think my first interpretation was correct. Again, is the co-injection of a whole plasmid with a restriction enzyme/Cas9 RNP really used in the field? Why? I am especially puzzled*

© 2025. Published by The Company of Biologists under the terms of the Creative Commons Attribution License (https://creativecommons.org/licenses/by/4.0/).

by the restriction enzyme; would it not wreak havoc in the genome? I see the recognition sequence is fairly long, but also that some degeneracy is tolerated… On my initial confusion: the authors should be much clearer in the results (not only in the methods) that e.g. a restriction enzyme is co-injected.
We are using standard techniques previously applied in the field across numerous knock-in studies and at this point any researcher in the zebrafish field should be familiar with these standard transgenesis and knock-in approaches. All RNPs and/or I-SceI meganuclease are mixed with templates and co-injected as a single mix, as has been done in numerous previous studies, the majority which are cited here. The use of I-SceI in co-injections has been used in numerous species to facilitate transgenesis and widely used in zebrafish prior to transposon-mediated approaches (examples - frog: PMID: 35135888; axolotl: PMID: 36272085; zebrafish, medaka: PMID: 12351173; mouse: PMID: 25250567). The I-SceI meganuclease has an 18-bp recognition site that is not found in most vertebrate genomes. In any case, additional details concerning I-SceI and more explicit descriptions of the templates are now included in the revised manuscript.

- About "In mammalian cells, the 3'-OH groups of single-stranded DNA donors […]" and after: I find it very difficult to follow the authors here; I assume the majority of readers would also get lost. Unfortunately, this makes it hard to judge the results as I'm not quite sure what is being asked. To point the authors to the issues:
-- "need to be unblocked": expects too much prior knowledge from the reader, I myself do not know exactly what is meant (i.e. what would "blocked" look like?).
-- Same for "3'-terminus of the donor might be extended […]. Thus, non-homologous nucleotides […]"; this logic will be impossible to understand for the majority of readers. I need to do a lot of guess work to complete the gaps. My current guess: the homology arm anneals to the complementary genomic strand and is polymerised by an endogenous DNA polymerase using the genome as template. So, if you have mismatches in the homology arm, it is like having mismatches in a PCR primer, this extension process will not work as well and HDR may be affected. Is this the logic? It is interesting but not trivial to understand, it requires much better explanation to be communicated, especially as the reader needs this knowledge to interpret the following results.
-- Took me multiple re-reads and a long stare at Fig. 1 to understand (I think) what are those "non-homologous nucleotides". I first thought the authors meant the A>G edit to inactivate the PAM of h3f3d? But then why plural "at these positions"… Now I think the authors mean the entire I-SceI site + the few nucleotides between the homology arm and the I-SceI site? Even if this is the correct interpretation, it requires much better explanation as it asks too much effort from the reader.
These points are all better explained and referenced in the revised manuscript.

-- "2'OMe modified primers": I understand this modification was mentioned before, but I doubt most readers will still have it in mind here. Perhaps a better wording would be like "synthesized primers…. As before, these included 2'-OMe modifications to reduce concatemerization".
We have now provided a bit more description of 2'OMe primers when they are first mentioned in the revised manuscript. Given that change and that we do not use any unmodified primers for template synthesis in the study, we trust that readers can remember the relevance of the 2'OMe here.

-- "universal PCR-amplified template": I have no idea what "universal" means here.
This has been changed in the revised manuscript.

-- "bearing non-homologous residues"; residue in my mind means amino acid? Correction: I see that it can be used for a nucleotide! I have never heard it used to say something else than amino acid though, so I would not use it to avoid confusing the reader, especially as "non-homologous nucleotide" is used just before.
The term "residue" is not specific to amino acids. Nonetheless, this has been changed in the revised manuscript.

-- "Since previous studies […]": are these the same v1 / v2 primers as introduced at the start ("Only v1 primers […]"), then I would not introduce them so early, only here.
-- "phosphorothioate linkages": need some explanation. What are they; why are they used here (I know they were mentioned before, but without any explanation either). I know what they are, and I think I can guess why they could be useful, but again, think about the PhD student in developmental biology!

© 2025. Published by The Company of Biologists under the terms of the Creative Commons Attribution License (https://creativecommons.org/licenses/by/4.0/).

Data and analysis of “version 2” PCR primers have been removed from the revised manuscript.

- Given the introduction sentences of this paragraph, I assume that the authors interpret the "five-fold increase in precise knock-in rate" as confirmation that the template has to be extended by this unknown endogenous polymerase? This hypothesis is raised (very superficially) but then the interpretation does not circle back to it.
This is now addressed in the Discussion section in the revised manuscript.

- Fig. 1A legend "Line located above or below sequence indicates strand, respectively", I do understand what this line! There is a sort of ruler in the middle of the sequences but no arrows or anything so that tells me the Forward or Reverse genomic directions. Also, "respectively" is used to match two things to two others (here the sentence says above & below both mean strand, so why respectively).
This has been modified in the revised manuscript.

- Fig. 1A: I would annotate the PAMs (e.g. bold is often used, or a frame). It will also make it easier to understand the PAM mutations (*).
This change has been made in the revised manuscript.

- Fig. 2A legend: "hey2 where a Mann-Whitney test was used"; I guess this is not very important as the p-value is non-significant anyways, but I found this a little surprising. If the authors think that the KI rates tend to follow a normal distribution (probably a reasonable assumption), why not use t-tests for all?
Mann-Whitney was used here as the distribution was not normal.

- Fig. 2: the legend does not say what is "sce", "uni", "mod". If I was a simple reader, I would probably have given up trying to understand the paper at this stage!; Fig. S1: legend does not explain the abbreviations "undig", "uninj". Again, I can guess, but the authors need to think about where they want to publish / who their target audience is.
These have been fixed in the revised manuscript.

- Fig. S3: the cartoon is OK but the legend is very bare-bone, one certainly cannot understand how the method works (e.g. what the UMIs are for) from this alone.
These points are addressed in the revised manuscript. Fig S3 is now part of Figure 2 where there is a more expansive description of library construction, which is otherwise covered in the Methods section.

- Fig. S5: I imagine that frequency is "out of all epitope insertions" or something like this? Is it not mentioned.
The original data in Fig. S5 are no longer included in the revised manuscript.

Some minor typos, wording, etc:
- Long/short-read sequencing with hyphen or not is not consistent (e.g. both "short-read" and "long read" in the abstract). I think the adjective should have a hyphen (e.g. long-read sequencing), but not if used as a noun ("long reads").
- Consistency of tenses (e.g. "we find…" and "we found…" in abstract).
- Consistency about double strand(ed) breaks / double-strand breaks.
*- "For PCR-amplified template, we used […]", I think *templates?*
- "We compared several different HDR templates", can delete "several".
- "nuclease activity by calculating insertion or deletion (indel)", the abbreviation "indel" was already introduced just above.
These have all been addressed in the revised manuscript.

Reviewer 2

Major issues:

1. Introduction : The concept of MMEJ is not discussed in this section, although multiple methods mentioned here utilize it to generate the knock-ins.

© 2025. Published by The Company of Biologists under the terms of the Creative Commons Attribution License (https://creativecommons.org/licenses/by/4.0/).

Mention of MMEJ is now included in the revised manuscript.

2. What is the significance of the 2'-O-Methyl-modified (2'OMe) ribose bases? Their predicted activity should be explained.
We have added further explanation of the end modification in the revised manuscript.

3. The significance and utility of the v1 vs. v2 primers should also be explained.
The version 2 primers have been removed from the revised manuscript.

4. The manuscript is somewhat confusing to follow regarding the various different constructs being generated and tested. Understanding would be greatly improved by including graphical summaries/schematics in each figure prior to the data in which that strategy is employed.
We have revised Figure 1 to include a better overview and schematic depiction of HDR templates being tested. The associated text has been likewise modified in the revised manuscript to better describe the constructs and templates.

5. The legend for Figure 2 is insufficiently detailed regarding the specific conditions tested. Moreover, the text related to Figure 2 is also not completely consistent with what is displayed in the figure, and again insufficient detail is provided. For example, the phrase "only at h3f3d and hey2" does not explain what occurred for the other genes in question and which nuclease is being employed. The data (text, figure and figure legend) need to be revised for completeness and clarity.
The Figure legend (now Figure 3) and associated text has been changed accordingly in the revised manuscript.

6. Total vs. precise knock ins should be defined.
This has been more clearly defined in the revised manuscript.

7. The universal vs. gene-specific nature of the modified primers was not introduced in the text. It is not clear from the manuscript what roles these RNA-based oligo parts play since they are separated by the I-SceI sites from the homology arms.
This is now made clearer in the revised manuscript.

8. Why did the authors only evaluate h3f3d with universal vs. gene-specific primers? It is unclear why all 4 combinations of universal vs homology-matched and v1 vs. v2 design versions were not tested. Why was there selected injection of data from one additional design combination but only in the germline transmission table? Additional genes should be tested to bolster the authors' conclusions around optimal conditions.
Given that we do not have complete data concerning v1 versus v2 primers, we have removed the v2 data from the revised manuscript. To demonstrate that our optimized parameters will have broader utility across other genes, we now provide data on 3 additional targets. In this case, we applied our optimized parameters and we assessed germline transmission and mosaicism without deep sequencing analysis, better mimicking “real-world” application of our results by prospective readers. We observe germline transmission of at least 20% for precise insertions across the 3 targets. These new findings are included in the revised manuscript. We believe that these new findings support our conclusion regarding optimal conditions for knock-in.

9. The authors state they did not observe any differences comparing templates amplified with v1 or v2 primers (Fig S7), however, there was a significant decrease in the 5' tag deletion rate for the v2 primers. Please address and provide further details.
As noted above, v2 data has been removed from the revised manuscript.

10. It would be highly advantageous to demonstrate the typical application of an epitope tagged line in terms of immunofluorescence and/or Western blotting. This would strengthen the practical utility of the method from the conceptual design to the direct biological application.
The revised manuscript now includes whole mount immunostaining of embryos bearing an epitope tagged allele of *prox1a*, underscoring the utility of this method.

Minor issues:

© 2025. Published by The Company of Biologists under the terms of the Creative Commons Attribution License (https://creativecommons.org/licenses/by/4.0/).

1. Introduction: Although technically in Medaka, a paper in eLife (https://elifesciences.org/articles/75050v2) provides an example where biotin-mSA interaction acts as the stimulatory factor for knock-in. It may be worth mentioning it here as well.
We would like to cite as many previous studies as possible. However, the revised manuscript has expanded somewhat and includes several new citations. Further, we have already cited a substantial number of relevant zebrafish paper where modified templates have been used. Therefore, we did not include this reference.

2. Results and Discussion: Please be more precise than "approximately 50 bp homology arms"? Was it always 50 bp and what were the reasons to choose other lengths?
I believe that one or two arms are 49 for no obvious reason, hence the use of "approximately".

3. Results and Discussion: The phrase "nearly complete loss of a restriction site" is not accurate due to heteroduplexes being resistant to digestion. The authors should modify to say something like "inactivation of digestion at an adjacent restriction site".
We have modified this description in the revised manuscript to refer to resistance to digestion rather than the loss of the site itself.

4. Results and Discussion: It is not clear exactly how and when the plasmid templates were cut to prepare the templates. It would be helpful to explain this at the first mention of "Plasmid-released" templates.
We mention co-injection of I-SceI or Cas9 more explicitly in the revised manuscript. It is also described as such in the Methods section.

5. Results and Discussion: The phrase "note that dose-response results were obtained using homology matched HDR templates, see below" implies that the relevant information was not introduced. The authors should edit the text to make such references unnecessary.
Conceptually, the dose-response results fit well with the comparison of nuclease activity and precise knock-in. However, the dose-response data were generated in a second set of experiments during which we tested homologous versus non-homologous ends. Given the significant amount of work involved in generating triplicate injections, library construction, etc. . . we did not perform a RNP dose response followed by sequencing using the original non-homologous templates. I agree that the presentation is a bit awkward, but the dose response data fit well here. To deal with this, we have moved the mention of the homology-matched template from the main text to the figure legend for the corresponding Supplementary Figure.

6. Results and Discussion: Table 1 and associated text -please include details about the nuclease used to improve readability.
This has been included in the revised text and Table.

7. Results and Discussion: Figure S2: Please describe abbreviations used across the x-axis in the figure legend.
This has been corrected in the revised manuscript.

8. Materials and Methods: The sentences "For templates released from plasmids, 50pg/µl of plasmid DNA was mixed with either 1 unit/µl I-SceI enzyme or 5µM Cas9 universal RNP and injected at 10 pg template DNA per embryo. For PCR-amplified templates, we injected 2.5 pg per embryo and all injection volumes were 2 nl." is not internally consistent. Plasmid DNA has incorrect units and its volume is not indicated. PCR template injection mix is formulated in a different way than the plasmid DNA. Please formulate them the same way.
This has been changed in the revised manuscript and is hopefully clearer now.

Reviewer 3: Reviewer comments for the authors:

- Introduction: "An important variable across targeted knock-in studies is the HDR template." This again comes up in the results part. It is unclear why the authors refer to HDR template and CRISPR nuclease as variables. Suggestion to rephrase "variable" with "component" or similar.
We use "variable" in these cases since <u>different</u> forms of template or CRISPR nucleases have been or are being applied. "Component" would not have the same connotation without adding further

© 2025. Published by The Company of Biologists under the terms of the Creative Commons Attribution License (https://creativecommons.org/licenses/by/4.0/).

descriptors. We are open to further suggestions, but for now have left the use of “variable” in the revised manuscript.

- Suggestion to shorten the introduction to make it more concise for readers.
Based on comments from the other reviewers, we have chosen to switch to the longer format (with permission from the editor). The introduction is approximately the same length but now includes a better description of and rationale for using short- versus long-read sequencing, as requested by reviewer 1.

- Could the authors include a comment on how affordable PacBio sequencing is at the moment for the readers?
The sequencing was done some time ago now, but in our core facility I believe it was between $1K-2K per flowcell. We have not checked on current pricing. My expectation is that the take-away for most readers will not be about using PacBio for a similar approach, but that they can have important points of emphasis for planning their knock-in designs to achieve efficient germline transmission.

- "Since previous studies using chemically modified templates had incorporated phosphorothioate linkages," Please provide reference.
The data on v2 modification has been removed from the revised manuscript.

- Refer to knockin earlier in the introduction.
This has been addressed in the revised manuscript.

- Clarify knockin template better in the results part of Fig. 1A.
We have made this much clearer in the revised manuscript, including incorporation of new schematics in Fig. 1.

- Methods and results part: please specify whether it was injected into the yolk or cell.
For any injection with DNA, it is important to inject only into the blastomere and avoid the yolk. This point has been added to the methods section in the revised manuscript.

- Please provide a sentence in the text on why epitope tags were only inserted 5' of the target genes.
This has been clarified in the revised manuscript.

- Clarify if tag is inserted in frame.
That was stated in the original manuscript and is retained in the revised manuscript.

- Please clarify "jackpot effects" for the broader readership in the results part.
This is now explained more clearly in the revised manuscript.

- We enjoyed reading the word monsters! But please clarify Monsters in FigS. 2. Please leave in Monsters but specify the meaning better and give examples of phenotypes describing monsters.
We have better explained that “monster” refers to non-specific morphological abnormalities in the revised manuscript.

- Suggestion to include for bullet point takeaways or graphical abstract.
We now provide an outline of considerations for knock-in design strategy. This is included with the revised mansuscript as File S1.

Second decision letter

MS ID#: dev.204571R1

MS TITLE: Identifying optimal conditions for precise knock-in of exogenous DNA into the zebrafish genome

© 2025. Published by The Company of Biologists under the terms of the Creative Commons Attribution License (https://creativecommons.org/licenses/by/4.0/).

AUTHORS: Sarah Oikemus; Kai Hu; Masahiro Shin; Feston Idrizi; Aliece Goodman-Khan; Amy Kolb; Krishna S. Ghanta; Atish Wagh; Jonathan Lee; Scot Wolfe; Lihua J. Zhu; Jonathan Watts; Nathan D. Lawson

Dear Nathan,

You will be pleased to see that the referees are mostly happy with your revisions and there are just some minor points for you to address/consider before we proceed to publication. The referees' comments are appended below, or you can access them online: please go to.

Reviewer 1

Most of my comments have been addressed. The revised manuscript is much clearer. The text could still be a bit more concise at times, but up to the authors if they want to work further on it.

I have a few remaining comments below:
- the main figures appear pixelated in my version. The supplementary ones look beter.
- line 87: "requires reliable sequence", I think "sequencing" or "sequencing reads"?
- In that sentence starting line 86, "to confirm precise knock-in events" and "to confirm precise HDR-mediated insertion" are redundant.
- line 117: I guess "random knock-in" refers to Thermes 2002. This terminology does not quite work as knock-in actually means targeted at a specific locus. Maybe replace knock-in by transgenesis?
- line 116 (and a more general comment the paper): I understand the authors' answer that this meganuclease approach is (or was) common but I am still slightly confused about the proposed mechanism for this "increase [in] knock-in rates" when using I-SceI. As the paper directly tests this approach, I think the authors should present briefly the rationale (i.e. why is it worth testing/why is it used), otherwise it sounds to the naÃ¯ve reader that both approaches should be identical (i.e. it should in theory not matter whether the HDR template is linear because it is a PCR amplicon or linear because it was cleaved from a plasmid). I am guessing it has to do with the ends being different?
- line 121: "by UgRNA", there is an error somewhere.
- line 213: "all loci for", error.
- legend Fig. 3, can the authors state what the abbreviations sce, uni, mod are.
- Discussion: about "limiting ligase-dependent repair events, such as non-homologous end joining". First, I'm assuming that every possible repair of a DNA break (including HDR) must involve a ligase at some point, but the sentence read naively states that HDR is somehow ligase-independent. Second, do the authors imply that NHEJ would directly ligate the injected template to the genome? There is no reference. I found a couple of papers in zebrafish making this claim but I am a bit confused as to how they would distinguish whether the insertion was made through HDR or NHEJ; wouldn't they both give the same outcome? It seems to me that one would need to first knock-out genes essential for NHEJ or HDR to make this claim. Schmid-Burgk 2016 seemed to have done just this, but it is in human cell cultures, while zebrafish embryos rarely use NHEJ. Finally, in the context of the paper, wouldn't these putative NHEJ-mediated knock-ins be beneficial, i.e. increase the total knock-in rates? If the total knock-in rate is indeed an addition of NHEJ-mediated knock-ins and HDR-mediated knock-ins, surely blocking the NHEJ events (with the chemical end-modifications) would decrease the rate of knock-in, which is not what was found.
- I am similarly confused by "extension of this sequence may force NHEJ-mediated repair". I am afraid I do not understand what the authors imply here. Which sequence? And why would it "force" NHEJ? NHEJ does not use a template, I understand there can be edge cases like the (putative) direct ligation of the template to the genome by NHEJ machinery, but talking about polymerase activity on the template in the context of NHEJ is confusing me.

Reviewer 2

SUMMARY OF THE ADVANCE MADE IN THIS PAPER AND ITS POTENTIAL SIGNIFICANCE TO THE FIELD

© 2025. Published by The Company of Biologists under the terms of the Creative Commons Attribution License (https://creativecommons.org/licenses/by/4.0/).

This manuscript provides a helpful addition to the growing literature for knock in gene editing in the zebrafish. Through a detailed comparative analysis, it clearly describes key considerations and optimizations to include when undertaking gene knock ins. The authors have responded well to the previous reviews, creating a much more complete and fulsome methods report. Moreover, the addition of the epitope-tagged prox1a together with functional validation using immunostaining, demonstrates the broad utility of the methodologies proposed.

SUGGESTIONS TO AUTHORS

1. Figure 3: "sce", "uni" and "mod" are still undefined. The authors define them in one of the supplemental figures, but they should be defined in the text and in the relevant legends.

2. The definition and distinction of universal vs. gene-specific primers remains unclear.

Reviewer 3

We enjoyed reading the revised version of this manuscript. The authors have clarified all concerns that were raised, with the exception of the following: If the word "variable" is used, we recommend stating "type of HDR template" as the variable described for clarity.

Second revision

Author response to reviewers' comments

Comments from the Reviewers:
Reviewer 1: Most of my comments have been addressed. The revised manuscript is much clearer. The text could still be a bit more concise at times, but up to the authors if they want to work further on it. I have a few remaining comments below:

- the main figures appear pixelated in my version. The supplementary ones look beter.
The PDF generated by Development has lo-res main figure images. Each respective figure page has an embedded link to allow download of the original hi-res TIFF file.

- line 87: "requires reliable sequence", I think "sequencing" or "sequencing reads"?
We have changed this to sequencing reads.

- In that sentence starting line 86, "to confirm precise knock-in events" and "to confirm precise HDR-mediated insertion" are redundant.
We have deleted “to confirm precise HDR-mediated insertion”.

- line 117: I guess "random knock-in" refers to Thermes 2002. This terminology does not quite work as knock-in actually means targeted at a specific locus. Maybe replace knock-in by transgenesis?
We have changed this to “random genomic insertion”.

- line 116 (and a more general comment the paper): I understand the authors' answer that this meganuclease approach is (or was) common but I am still slightly confused about the proposed mechanism for this "increase [in] knock-in rates" when using I-SceI. As the paper directly tests this approach, I think the authors should present briefly the rationale (i.e. why is it worth testing/why is it used), otherwise it sounds to the naïve reader that both approaches should be identical (i.e. it should in theory not matter whether the HDR template is linear because it is a PCR amplicon or linear because it was cleaved from a plasmid). I am guessing it has to do with the ends being different?
We have re-written the first paragraph of the Results section to better explain the rationale for choosing the HDR templates.

© 2025. Published by The Company of Biologists under the terms of the Creative Commons Attribution License (https://creativecommons.org/licenses/by/4.0/).

- line 121: "by UgRNA", there is an error somewhere.
This section has been rewritten.

- line 213: "all loci for", error.
This has been corrected.

- legend Fig. 3, can the authors state what the abbreviations sce, uni, mod are.
This has been corrected.

- Discussion: about "limiting ligase-dependent repair events, such as non-homologous end joining". First, I'm assuming that every possible repair of a DNA break (including HDR) must involve a ligase at some point, but the sentence read naively states that HDR is somehow ligase-independent. Second, do the authors imply that NHEJ would directly ligate the injected template to the genome? There is no reference. I found a couple of papers in zebrafish making this claim but I am a bit confused as to how they would distinguish whether the insertion was made through HDR or NHEJ; wouldn't they both give the same outcome? It seems to me that one would need to first knock-out genes essential for NHEJ or HDR to make this claim. Schmid-Burgk 2016 seemed to have done just this, but it is in human cell cultures, while zebrafish embryos rarely use NHEJ. Finally, in the context of the paper, wouldn't these putative NHEJ-mediated knock-ins be beneficial, i.e. increase the total knock-in rates? If the total knock-in rate is indeed an addition of NHEJ-mediated knock-ins and HDR-mediated knock-ins, surely blocking the NHEJ events (with the chemical end-modifications) would decrease the rate of knock-in, which is not what was found.
We have removed that phrase from the Discussion.

- I am similarly confused by "extension of this sequence may force NHEJ-mediated repair". I am afraid I do not understand what the authors imply here. Which sequence? And why would it "force" NHEJ? NHEJ does not use a template, I understand there can be edge cases like the (putative) direct ligation of the template to the genome by NHEJ machinery, but talking about polymerase activity on the template in the context of NHEJ is confusing me.
We have removed reference to NHEJ. That sentence now reads: “Since homologous recombination typically requires 3’ annealing to prime repair strand extension, non-homologous sequences could hinder this process leading to alternative repair mechanisms that incorporate unmatched sequence at the target.”

Reviewer 2: SUMMARY OF THE ADVANCE MADE IN THIS PAPER AND ITS POTENTIAL SIGNIFICANCE TO THE FIELD

This manuscript provides a helpful addition to the growing literature for knock in gene editing in the zebrafish. Through a detailed comparative analysis, it clearly describes key considerations and optimizations to include when undertaking gene knock ins. The authors have responded well to the previous reviews, creating a much more complete and fulsome methods report. Moreover, the addition of the epitope-tagged prox1a together with functional validation using immunostaining, demonstrates the broad utility of the methodologies proposed.

SUGGESTIONS TO AUTHORS

1. Figure 3: "sce", "uni" and "mod" are still undefined. The authors define them in one of the supplemental figures, but they should be defined in the text and in the relevant legends.
This has been addressed. We now include reference to these abbreviations at their first mention in the text, as well as in the Figure 3 legend. To avoid confusion with other uses of the word “universal”, we refer to the Cas9-digested plasmids by the abbreviation “Ug” (for UgRNA).

2. The definition and distinction of universal vs. gene-specific primers remains unclear.
We have added additional text to better describe the universal primers in the library construction. We have otherwise tried to limit the use of “universal” in other parts of the manuscript, except when initially referring to the universal gRNA.

© 2025. Published by The Company of Biologists under the terms of the Creative Commons Attribution License (https://creativecommons.org/licenses/by/4.0/).

Reviewer 3: We enjoyed reading the revised version of this manuscript. The authors have clarified all concerns that were raised, with the exception of the following: If the word "variable" is used, we recommend stating "type of HDR template" as the variable described for clarity.
There are 4 instances of “variable” in the manuscript. Two are used as verbs. The other two cases, which we believe are well-defined (definitions noted in italics below), are:

Line 41, “A common variable across targeted knock-in studies is the *type of HDR template*.”

Lines 323-326 “. .the *distance between the DSB and the point of insertion for exogenous sequence* was an important factor. . . zebrafish appear more sensitive to this variable than human cells. .”

Third decision letter

MS ID#: dev.204571R2

MS TITLE: Identifying optimal conditions for precise knock-in of exogenous DNA into the zebrafish genome

AUTHORS: Sarah Oikemus; Kai Hu; Masahiro Shin; Feston Idrizi; Aliece Goodman-Khan; Amy Kolb; Krishna S. Ghanta; Atish Wagh; Jonathan Lee; Scot Wolfe; Lihua J. Zhu; Jonathan Watts; Nathan D. Lawson
ARTICLE TYPE: Techniques and Resources Article

Dear Nathan,

I am happy to tell you that your manuscript has been accepted for publication in Development, pending our standard publication integrity checks. It was accepted on 14 May 2025.

© 2025. Published by The Company of Biologists under the terms of the Creative Commons Attribution License (https://creativecommons.org/licenses/by/4.0/).
